# Supplementary material for: Targeting Group 3 Medulloblastoma by the Anti-PRUNE-1 and Anti-LSD1/KDM1A Epigenetic Molecules
Source: Int J Mol Sci. 2024 Mar 31;25(7):3917. doi: 10.3390/ijms25073917 (PMC11011515; doi:10.3390/ijms25073917)
Supplement: Supplementary file 1 [file ijms-25-03917-s001.zip › Supp. Figure Legends.pdf]

Supplemental Figures.

Supplemental Figure 1.

A. Genome browser screenshots showing accumulation of normalized LSD1 signal on the transcription start sites (TSS) of Prune-1 transcripts in in human neuroblastoma cells SH-SY5Y (Laurent et al., 2015).

B. Genome browser screenshots showing accumulation of normalized LSD1 signal on the transcription start sites (TSS) of Prune-1 transcripts murine MB cells (Lee et al., 2019).

C. Genome browser screenshots showing accumulation of normalized LSD1 signal on the transcription start sites (TSS) of Otx2 transcripts in in human pancreatic progenitors (Vinckier et al., 2020) and human SCLC cells (Mohammad et al., 2015).

D. Genome browser screenshots showing accumulation of normalized LSD1 signal on the transcription start sites (TSS) of Otx2 transcripts in ES murine cells (Cao et a., 2018; Nair et al., 2012).

E. Genome browser screenshots showing accumulation of normalized LSD1 signal on the transcription start sites (TSS) of NME1-2 transcripts in murine medulloblastoma cells (Lee et al., 2019; Wang et al. 2015).

Supplemental Figure 2.

A. Real-time cell proliferation analyses for the Cell Index. D425-Med were plated and treated with the indicated concentrations (escalating doses) of TCP; with vehicle-treated cells were the negative control. Impedance was measured every 2 min over 24 hours. The graph showing "cell index" was generated using Graph Pad Prism 9.

B. Quantification of mRNA abundance relative to untreated control cells (fold change) for PRUNE-1 gene. RT-PCR analysis of RNA extracted from D425-Med cells treated with 1mM TCP. Data are means ±SD. * p <0.05, **p<0.1 (unpaired two-tailed student's t-tests; N = 3).

C. Quantification of mRNA abundance relative to untreated control cells (fold change) for PRUNE-1 and NRG1 genes. RT-PCR analysis of RNA extracted from D283-Med cells treated with 1mM TCP. Data are means ±SD. * p <0.05, **p<0.1 (unpaired two-tailed student's t-tests; N = 3).

Supplemental Figure 3.

A. Real-time cell proliferation analyses for the Cell Index. D425-Med were plated and treated with the indicated concentrations (escalating doses) of SP-2577; with vehicle-treated cells were the negative control. Impedance was measured every 2 min over 24 hours. The graph showing "cell index" was generated using Graph Pad Prism 9.

B. IC_{50} (R^2 9.3894e-1) value was calculated through nonlinear regression analysis performed with Graph Pad Prism 9 ([inhibitor] vs. response [three parameters]) for SP-2577. The graph showing "cell index" was generated using Graph Pad Prism 9

C. Real-time cell proliferation analyses for the Cell Index (i.e., the cell-sensor impedance was expressed every two minutes as a unit called "Cell Index"). D283-Med were plated and treated with the indicated concentrations (escalating doses) of SP-2577; with vehicle-treated cells were the

negative control. Impedance was measured every 2 min over 24 hours. The graph showing “cell index” was generated using Graph Pad Prism 9.

D. IC_{50} (R^2 9e-1) value was calculated through nonlinear regression analysis performed with Graph Pad Prism 9 ([inhibitor] vs. response [three parameters]) for SP-2577.

Supplemental Figure 4.

A. Multidimensional scaling of the comparative PCA analysis on RNA-seq replicates data.

B. Read counts of Prune-1 in th different conditions of RNA-seq experiments. Its down-regulation in treated cells was used as control of the treatments efficacy.

C. Volcano plot representing the differentially expressed genes in cells treated with AA7.1 compared to vehicle cells.

D. Volcano plot representing the differentially expressed genes in cells treated with SP-2577 compared to vehicle cells.

E. Venn diagram representation of common up-regulated in the three treatment conditions (AA7.1, SP-2577 and the combination of two) compared to vehicle cells.

F. Venn diagram representation of common down-regulated genes in the three treatment conditions (AA7.1, SP-2577 and the combination of two) compared to vehicle cells.
