# Supplementary material for: Targeting Group 3 Medulloblastoma by the Anti-PRUNE-1 and Anti-LSD1/KDM1A Epigenetic Molecules
Source: Int J Mol Sci. 2024 Mar 31;25(7):3917. doi: 10.3390/ijms25073917 (PMC11011515; doi:10.3390/ijms25073917)
Supplement: Supplementary file 1 [file ijms-25-03917-s001.zip › Supp. Tables.pdf]

Supplemental Tables

Supp. Table S1. Nonsynonymous SNV found through WES analysis on DNA extracted from biopsy of patient affected by Group3 MB.

Gene Symbol	ID transcript	Exon	DNA alteration	Protein alteration	Variant
**ACSF2**	NM_001288971	exon4	c.C290A	p.P97H	nonsynonymous SNV
**AHCYL1**	NM_001242673	exon7	c.C538A	p.L180M	nonsynonymous SNV
**ALPI**	NM_001631	exon5	c.G506T	p.R169L	nonsynonymous SNV
**ANKLE1**	NM_001278443	exon6	c.G1223T	p.G408V	nonsynonymous SNV
**BACH1**	NM_001186	exon4	c.C1675A	p.H559N	nonsynonymous SNV
**BEST2**	NM_017682	exon3	c.C218T	p.A73V	nonsynonymous SNV
**CACNA1G**	NM_001256324	exon12	c.G2684T	p.R895L	nonsynonymous SNV
**CCDC115**	NM_001321118	exon5	c.G465T	p.W155C	nonsynonymous SNV
**CDH5**	NM_001795	exon12	c.G1959T	p.M653I	nonsynonymous SNV
**CEP19**	NM_032898	exon3	c.G247T	p.G83W	nonsynonymous SNV
**CISH**	NM_145071	exon3	c.G683T	p.R228L	nonsynonymous SNV
**CITED1**	NM_001144886	exon3	c.G496T	p.G166W	nonsynonymous SNV
**COL13A1**	NM_001368896	exon24	c.C1307A	p.P436Q	nonsynonymous SNV
**COL16A1**	NM_001856	exon52	c.G3314T	p.G1105V	nonsynonymous SNV
**COL24A1**	NM_001349955	exon50	c.G2086T	p.G696C	nonsynonymous SNV
**COL5A1**	NM_000093	exon23	c.C2167A	p.Q723K	nonsynonymous SNV
**CRYM**	NM_001888	exon3	c.G169T	p.G57C	nonsynonymous SNV
**EPX**	NM_000502	exon12	c.G1953T	p.W651C	nonsynonymous SNV
**FAM102B**	NM_001010883	exon4	c.C343A	p.R115S	nonsynonymous SNV
**FAM126A**	NM_001363466	exon8	c.G727T	p.G243W	nonsynonymous SNV
**FAM186B**	NM_032130	exon2	c.G321T	p.W107C	nonsynonymous SNV
**FLNA**	NM_001456	exon47	c.G7754T	p.G2585V	nonsynonymous SNV
**GFM1**	NM_024996	exon15	c.C1862A	p.S621Y	nonsynonymous SNV
**GMPPA**	NM_013335	exon5	c.C263A	p.P88H	nonsynonymous SNV
**GOLGA7B**	NM_001010917	exon2	c.G137T	p.R46L	nonsynonymous SNV
**GPATCH1**	NM_018025	exon3	c.G286T	p.D96Y	nonsynonymous SNV

**HID1**	NM_030630	exon2	c.G176T	p.R59L	nonsynonymous SNV
**KDM4A**	NM_014663	exon19	c.G2815T	p.D939Y	nonsynonymous SNV
**KIAA0355**	NM_014686	exon8	c.G1273T	p.D425Y	nonsynonymous SNV
**LRP1**	NM_002332	exon25	c.G4079T	p.W1360L	nonsynonymous SNV
**LRRC8D**	NM_001134479	exon3	c.C2372A	p.P791Q	nonsynonymous SNV
**LRRFIP2**	NM_001348311	exon9	c.C716A	p.S239X	stopgain
**MTHFD2L**	NM_001144978	exon3	c.T428G	p.I143R	nonsynonymous SNV
**MYO7A**	NM_000260	exon23	c.C2870A	p.P957Q	nonsynonymous SNV
**NR5A1**	NM_004959	exon6	c.G1049T	p.R350L	nonsynonymous SNV
**NUP155**	NM_001278312	exon30	c.G3377T	p.G1126V	nonsynonymous SNV
**OPN1SW**	NM_001708	exon2	c.G391T	p.E131X	stopgain
**PAX6**	NM_001368902	exon3	c.G85T	p.G29W	nonsynonymous SNV
**PDE1B**	NM_001315535	exon8	c.C509A	p.P170Q	nonsynonymous SNV
**PDLIM1**	NM_020992	exon2	c.248+1G>A		splicing
**PITHD1**	NM_020362	exon3	c.C305A	p.P102H	nonsynonymous SNV
**PKHD1L1**	NM_177531	exon37	c.A4747T	p.T1583S	nonsynonymous SNV
**PLPP1**	NM_003711	exon3	c.G463T	p.G155W	nonsynonymous SNV
**PTPRN**	NM_001199763	exon18	c.T2573A	p.L858Q	nonsynonymous SNV
**PTPRS**	NM_130853	exon26	c.C4117A	p.L1373M	nonsynonymous SNV
**RIPK4**	NM_020639	exon2	c.G382T	p.G128C	nonsynonymous SNV
**RPAP2**	NM_024813	exon9	c.1456-1G>A		splicing
**SCN11A**	NM_014139	exon1	c.C208A	p.P70T	nonsynonymous SNV
**SLC25A36**	NM_001104647	exon6	c.C737A	p.P246Q	nonsynonymous SNV
**SLC25A46**	NM_001303249	exon1	c.C17A	p.P6Q	nonsynonymous SNV
**SMG1**	NM_015092	exon44	c.G7194T	p.Q2398H	nonsynonymous SNV
**STOM**	NM_001270526	exon6	c.G555T	p.W185C	nonsynonymous SNV
**SVEP1**	NM_153366	exon31	c.G5071T	p.G1691W	nonsynonymous SNV
**TCF7L2**	NM_001349870	exon3	c.C302A	p.P101Q	nonsynonymous SNV
**TMPRSS4**	NM_001173552	exon7	c.C490A	p.R164S	nonsynonymous SNV
**TRPC7**	NM_001167576	exon2	c.G314T	p.R105L	nonsynonymous SNV

Supp. Table S2. Statistically up-regulated genes fc.COMB/CTR > 5, implicated in neuronal differentiation.

**Gene name**	**fc.COMB/CTR**	**bh.pval**	**Function**
**AATK**	9,807387	2,85E-08	Serine/threonine-protein kinase LMTK1; May be involved in neuronal differentiation. Belongs to the protein kinase superfamily. Tyr protein kinase family.
**ACHE**	11,73868	1,45E-14	Acetylcholinesterase; Terminates signal transduction at the neuromuscular junction by rapid hydrolysis of the acetylcholine released into the synaptic cleft. Role in neuronal apoptosis. Belongs to the type-B carboxylesterase/lipase family.
**ADM**	5,967177	5,4E-22	Proadrenomedullin N-20 terminal peptide; AM and PAMP are potent hypotensive and vasodilatator agents. Numerous actions have been reported most related to the physiologic control of fluid and electrolyte homeostasis.
**ARC**	20,61495	4,53E-11	Activity-regulated cytoskeleton-associated protein; Master regulator of synaptic plasticity that self-assembles into virion-like capsids that encapsulate RNAs and mediate intercellular RNA transfer in the nervous system. ARC protein is released from neurons in extracellular vesicles that mediate the transfer of ARC mRNA into new target cells, where ARC mRNA can undergo activity-dependent translation. ARC capsids are endocytosed and are able to transfer ARC mRNA into the cytoplasm of neurons.
**BAIAP2**	5,585796	3,53E-17	Brain-specific angiogenesis inhibitor 1-associated protein 2; Adapter protein that links membrane-bound small G-proteins to cytoplasmic effector proteins. Necessary for CDC42-mediated reorganization of the actin cytoskeleton and for RAC1-mediated membrane ruffling. Involved in the regulation of the actin cytoskeleton by WASF family members and the Arp2/3 complex. Plays a role in neurite growth.
**BHLHE41**	10,22933	7,19E-31	Class E basic helix-loop-helix protein 41; Transcriptional repressor involved in the regulation of the circadian rhythm by negatively regulating the activity of the clock genes and clock-controlled genes. Acts as the negative limb of a novel autoregulatory feedback loop (DEC loop) which differs from the one formed by the PER and CRY transcriptional repressors (PER/CRY loop).
**BNIP3**	8,226858	3,52E-30	BCL2/adenovirus E1B 19 kDa protein-interacting protein 3; Apoptosis-inducing protein that can overcome BCL2 suppression. May play a role in repartitioning calcium between the two major intracellular calcium stores in association with BCL2.
**BRSK2**	8,900804	6,12E-14	Serine/threonine-protein kinase BRSK2; Serine/threonine-protein kinase that plays a key role in polarization of neurons and axonogenesis, cell cycle progress. Acts as a key regulator of polarization of cortical neurons, probably by mediating phosphorylation of microtubule-associated proteins such as MAPT/TAU at 'Thr-529' and 'Ser-579'. Also regulates neuron polarization by mediating phosphorylation of WEE1 at 'Ser-642' in postmitotic neurons
**C3orf70**	36,17561	9,61E-37	May play a role in neuronal and neurobehavioral development.
**CAMK4**	7,380502	0,000332	Calcium/calmodulin-dependent protein kinase type IV; Calcium/calmodulin-dependent protein kinase that operates in the calcium-triggered CaMKK-CaMK4 signaling cascade and regulates, mainly by phosphorylation, the activity of several transcription activators, such as CREB1, MEF2D, JUN and RORA, which play pivotal roles in immune response, inflammation, and memory consolidation.
**CELSR1**	5,265506	1,51E-09	Cadherin EGF LAG seven-pass G-type receptor 1; Receptor that may have an important role in cell/cell signaling during nervous system formation.
**CHAC1**	8,413587	1,17E-15	Glutathione-specific gamma-glutamylcyclotransferase 1; Catalyzes the cleavage of glutathione into 5-oxo-L-proline and a Cys-Gly dipeptide. Acts specifically on glutathione, but not on other gamma-glutamyl peptides.
**CHRM2**	18,27706	1,86E-07	Muscarinic acetylcholine receptor M2; The muscarinic acetylcholine receptor mediates various cellular responses, including inhibition of adenylate cyclase, breakdown of phosphoinositides and modulation of potassium channels through the action of G proteins. Primary transducing effect is adenylate cyclase inhibition.
**CLCF1**	7,880602	3,48E-21	Cardiotrophin-like cytokine factor 1; In complex with CRLF1, forms a heterodimeric neurotropic cytokine that plays a crucial role during neuronal development

**CLDN3**	151,2257	5,46E-62	Claudin-3; Plays a major role in tight junction-specific obliteration of the intercellular space, through calcium-independent cell-adhesion activity; Belongs to the claudin family.
**CPNE5**	18,81697	3,19E-22	Copine-5; Probable calcium-dependent phospholipid-binding protein that may play a role in calcium-mediated intracellular processes
**CYFIP2**	5,841267	1,08E-19	Cytoplasmic FMR1-interacting protein 2; Involved in T-cell adhesion and p53/TP53-dependent induction of apoptosis. Does not bind RNA. As component of the WAVE1 complex, required for BDNF-NTRK2 endocytic trafficking and signaling from early endosomes
**DDIT4**	6,308914	3,85E-24	DNA damage-inducible transcript 4 protein; Regulates cell growth, proliferation and survival via inhibition of the activity of the mammalian target of rapamycin complex 1 (mTORC1). Inhibition of mTORC1 is mediated by a pathway that involves DDIT4/REDD1, AKT1, the TSC1-TSC2 complex and the GTPase RHEB. Plays an important role in responses to cellular energy levels and cellular stress.
**DGKG**	5,487889	0,000104	Diacylglycerol kinase gamma; Reverses the normal flow of glycerolipid biosynthesis by phosphorylating diacylglycerol back to phosphatidic acid.
**DKK1**	10,04935	2,46E-20	Dickkopf-related protein 1; Antagonizes canonical Wnt signaling by inhibiting LRP5/6 interaction with Wnt and by forming a ternary complex with the transmembrane protein KREMEN that promotes internalization of LRP5/6.
**DPYSL4**	169,8284	1,1E-110	Dihydropyrimidinase-related protein 4; Necessary for signaling by class 3 semaphorins and subsequent remodeling of the cytoskeleton. Plays a role in axon guidance, neuronal growth cone collapse and cell migration. Belongs to the metallo-dependent hydrolases superfamily. Hydantoinase/dihydropyrimidinase family
**EFNA3**	8,307754	3,12E-17	Ephrin-A3; Cell surface GPI-bound ligand for Eph receptors, a family of receptor tyrosine kinases which are crucial for migration, repulsion and adhesion during neuronal, vascular and epithelial development.
**EGR2**	11,23813	3,77E-12	E3 SUMO-protein ligase EGR2; Sequence-specific DNA-binding transcription factor. Binds to two specific DNA sites located in the promoter region of HOXA4. Belongs to the EGR C2H2-type zinc-finger protein family.
**EOMES**	5,925741	0,003359	Eomesodermin homolog; Functions as a transcriptional activator playing a crucial role during development. Functions in trophoblast differentiation and later in gastrulation, regulating both mesoderm delamination and endoderm specification. Plays a role in brain development being required for the specification and the proliferation of the intermediate progenitor cells and their progeny in the cerebral cortex. Also involved in the differentiation of CD8+ T-cells.
**EPHB1**	14,38829	1,27E-09	Ephrin type-B receptor 1; Receptor tyrosine kinase which binds promiscuously transmembrane ephrin-B family ligands residing on adjacent cells, leading to contact-dependent bidirectional signaling into neighboring cells. The signaling pathway downstream of the receptor is referred to as forward signaling while the signaling pathway downstream of the ephrin ligand is referred to as reverse signaling.
**EPOP**	5,254112	0,002044	Elongin BC and Polycomb repressive complex 2-associated protein; Scaffold protein that serves as a bridging partner between the PRC2/EED-EZH2 complex and the elongin BC complex: required to fine- tune the transcriptional status of Polycomb group (PcG) target genes in embryonic stem cells (ESCs).
**FGF11**	11,68187	1,2E-34	Fibroblast growth factor 11; Probably involved in nervous system development and function; Belongs to the heparin-binding growth factors family.
**FZD1**	5,199823	6,63E-16	Frizzled-1; Receptor for Wnt proteins. Activated by WNT3A, WNT3, WNT1 and to a lesser extent WNT2, but apparently not by WNT4, WNT5A, WNT5B, WNT6, WNT7A or WNT7B.
**FZD3**	10,18818	5,26E-15	Frizzled-3; Receptor for Wnt proteins. Most of frizzled receptors are coupled to the beta-catenin canonical signaling pathway, which leads to the activation of disheveled proteins, inhibition of GSK-3 kinase, nuclear accumulation of beta-catenin and activation of Wnt target genes.
**FZD7**	5,929052	4,11E-14	Frizzled-7; Receptor for Wnt proteins. Most frizzled receptors are coupled to the beta-catenin canonical signaling pathway, which leads to the activation of disheveled proteins, inhibition of GSK-3 kinase, nuclear accumulation of beta-catenin and activation of Wnt target genes. A second signaling pathway involving PKC and calcium fluxes has been seen for some family members, but it is not yet clear if it represents a distinct pathway or if it can be integrated in the canonical pathway, as PKC seems to be required for Wnt-mediated inactivation of GSK-3 kinase.
**HLX**	5,135061	3,73E-08	H2.0-like homeobox protein; Transcription factor required for TBX21/T-bet-dependent maturation of Th1 cells as well as maintenance of Th1-specific gene expression. Involved in embryogenesis and hematopoiesis.

**INA**	30,75094	4,82E-22	Alpha-internexin; Class-IV neuronal intermediate filament that is able to self- assemble. It is involved in the morphogenesis of neurons. It may form an independent structural network without the involvement of other neurofilaments or it may cooperate with NF-L to form the filamentous backbone to which NF-M and NF-H attach to form the cross-bridges.
**IRX5**	6,077889	4,44E-09	Iroquois-class homeodomain protein IRX-5; Establishes the cardiac repolarization gradient by its repressive actions on the KCND2 potassium-channel gene. Required for retinal cone bipolar cell differentiation.
**JAG2**	7,117793	1,37E-13	Protein jagged-2; Putative Notch ligand involved in the mediation of Notch signaling. Involved in limb development
**KCTD11**	14,11553	6E-30	BTB/POZ domain-containing protein KCTD11; Plays a role as a marker and a regulator of neuronal differentiation; Up-regulated by a variety of neurogenic signals, such as retinoic acid, epidermal growth factor/EGF and NGFB/nerve growth factor. Induces apoptosis, growth arrest and the expression of cyclin- dependent kinase inhibitor CDKN1B. Plays a role as a tumor repressor and inhibits cell growth and tumorigenicity of medulloblastoma (MDB).
**KDM2B**	6,212372	1,67E-16	Lysine-specific demethylase 2B; Histone demethylase that demethylates 'Lys-4' and 'Lys-36' of histone H3, thereby playing a central role in histone code. Preferentially demethylates trimethylated H3 'Lys-4' and dimethylated H3 'Lys-36' residue while it has weak or no activity for mono- and tri-methylated H3 'Lys-36'. Preferentially binds the transcribed region of ribosomal RNA and represses the transcription of ribosomal RNA genes which inhibits cell growth and proliferation.
**KDM7A**	13,82814	1,49E-37	Lysine-specific demethylase 7A; Histone demethylase required for brain development. Specifically demethylates dimethylated 'Lys-9' and 'Lys-27' (H3K9me2 and H3K27me2, respectively) of histone H3 and monomethylated histone H4 'Lys-20' residue (H4K20Me1), thereby playing a central role in histone code. Specifically binds trimethylated 'Lys-4' of histone H3 (H3K4me3), affecting histone demethylase specificity: in presence of H3K4me3, it has no demethylase activity toward H3K9me2, while it has high activity toward H3K27me2. Demethylates H3K9me2 in absence of H3K4me3.
**KLF4**	5,554976	2,19E-13	Krueppel-like factor 4; Transcription factor; can act both as activator and as repressor. Binds the 5'-CACCC-3' core sequence. Binds to the promoter region of its own gene and can activate its own transcription. Regulates the expression of key transcription factors during embryonic development. Plays an important role in maintaining embryonic stem cells, and in preventing their differentiation.
**LIF**	6,519591	7,83E-11	Leukemia inhibitory factor; LIF has the capacity to induce terminal differentiation in leukemic cells. Its activities include the induction of hematopoietic differentiation in normal and myeloid leukemia cells, the induction of neuronal cell differentiation.
**MAPT**	10,95531	1,36E-16	Microtubule-associated protein tau; Promotes microtubule assembly and stability, and might be involved in the establishment and maintenance of neuronal polarity. The C-terminus binds axonal microtubules while the N-terminus binds neural plasma membrane components, suggesting that tau functions as a linker protein between both. Axonal polarity is predetermined by TAU/MAPT localization (in the neuronal cell) in the domain of the cell body defined by the centrosome.
**MFSD2A**	22,37531	2,34E-18	Sodium-dependent lysophosphatidylcholine symporter 1; Sodium-dependent lysophosphatidylcholine (LPC) symporter, which plays an essential role for blood-brain barrier formation and function (By similarity). Specifically expressed in endothelium of the blood-brain barrier of micro-vessels and transports LPC into the brain.
**NCAM1**	6,116266	1,63E-07	eural cell adhesion molecule 1; This protein is a cell adhesion molecule involved in neuron- neuron adhesion, neurite fasciculation, outgrowth of neurites.
**NEDD4**	5,329726	2,91E-12	E3 ubiquitin-protein ligase NEDD4; E3 ubiquitin-protein ligase which accepts ubiquitin from an E2 ubiquitin-conjugating enzyme in the form of a thioester and then directly transfers the ubiquitin to targeted substrates.
**NEDD4L**	6,075925	1,73E-22	E3 ubiquitin-protein ligase NEDD4-like; E3 ubiquitin-protein ligase which accepts ubiquitin from an E2 ubiquitin-conjugating enzyme in the form of a thioester and then directly transfers the ubiquitin to targeted substrates. Inhibits TGF- beta signaling by triggering SMAD2 and TGFBR1 ubiquitination and proteasome-dependent degradation.
**NEFL**	15,615	5,96E-47	Neurofilament light polypeptide; Neurofilaments usually contain three intermediate filament proteins: L, M, and H which are involved in the maintenance of neuronal caliber.
**NGEF**	6,120873	2,23E-06	Ephexin-1; Acts as a guanine nucleotide exchange factor (GEF) which differentially activates the GTPases RHOA, RAC1 and CDC42. Plays a role in axon guidance regulating ephrin-induced growth cone collapse and dendritic spine morphogenesis. Upon activation by ephrin through EPHA4, the GEF activity switches toward RHOA resulting in its activation. Activated RHOA promotes cone retraction at the expense of RAC1- and CDC42-stimulated growth cone extension

**NOTCH3**	5,179835	5,15E-18	Neurogenic locus notch homolog protein 3; Functions as a receptor for membrane-bound ligands Jagged1, Jagged2 and Delta1 to regulate cell-fate determination. Upon ligand activation through the released notch intracellular domain (NICD) it forms a transcriptional activator complex with RBPJ/RBPSUH and activates genes of the enhancer of split locus.
**NPTX1**	12,14229	3,09E-11	Neuronal pentraxin-1; May be involved in mediating uptake of synaptic material during synapse remodeling or in mediating the synaptic clustering of AMPA glutamate receptors at a subset of excitatory synapses.
**NTM**	7,944967	2,23E-13	Neurotrimin; Neural cell adhesion molecule; Belongs to the immunoglobulin superfamily. IgLON family.
**PKD1**	5,300227	7,89E-18	Polycystin-1; Component of a heteromeric calcium-permeable ion channel formed by PKD1 and PKD2 that is activated by interaction between PKD1 and a Wnt family member, such as WNT3A and WNT9B. Both PKD1 and PKD2 are required for channel activity. Involved in renal tubulogenesis.
**PLK5**	5,616208	6,01E-07	Inactive serine/threonine-protein kinase PLK5; Inactive serine/threonine-protein kinase that plays a role in cell cycle progression and neuronal differentiation. Belongs to the protein kinase superfamily. Ser/Thr protein kinase family. CDC5/Polo subfamily.
**PLXNB3**	6,678516	2,1E-08	Plexin-B3; Receptor for SEMA5A that plays a role in axon guidance, invasive growth and cell migration. Stimulates neurite outgrowth and mediates Ca(2+)/Mg(2+)-dependent cell aggregation. In glioma cells, SEMA5A stimulation of PLXNB3 results in the disassembly of F-actin stress fibers, disruption of focal adhesions and cellular collapse as well as inhibition of cell migration and invasion through ARHGDIA- mediated inactivation of RAC1; Belongs to the plexin family.
**PPP2R5B**	5,010516	1,33E-17	Serine/threonine-protein phosphatase 2A 56 kDa regulatory subunit beta isoform; As the regulatory component of the serine/threonine-protein phosphatase 2A (PP2A) holoenzyme, modulates substrate specificity, subcellular localization, and responsiveness to phosphorylation. The phosphorylated form mediates the interaction between PP2A and AKT1, leading to AKT1 dephosphorylation.
**PTPRF**	13,99128	1,07E-38	Receptor-type tyrosine-protein phosphatase F; Possible cell adhesion receptor. It possesses an intrinsic protein tyrosine phosphatase activity (PTPase) and dephosphorylates EPHA2 regulating its activity.
**RAP1GAP**	7,129759	5,1E-06	Rap1 GTPase-activating protein 1; GTPase activator for the nuclear Ras-related regulatory protein RAP-1A (KREV-1), converting it to the putatively inactive GDP- bound state.
**RGS2**	6,591758	1,64E-21	Regulator of G-protein signaling 2; Regulates G protein-coupled receptor signaling cascades. Inhibits signal transduction by increasing the GTPase activity of G protein alpha subunits, thereby driving them into their inactive GDP- bound form. It is involved in the negative regulation of the angiotensin-activated signaling pathway.
**RORA**	11,05511	2,88E-38	Nuclear receptor ROR-alpha; Nuclear receptor that binds DNA as a monomer to ROR response elements (RORE) containing a single core motif half-site 5'-AGGTCA-3' preceded by a short A-T-rich sequence. Key regulator of embryonic development, cellular differentiation, immunity, circadian rhythm as well as lipid, steroid, xenobiotics and glucose metabolism.
**RTN4R**	4,801807	6,68E-05	Reticulon-4 receptor; Receptor for RTN4, OMG and MAG. Functions as receptor for the sialylated gangliosides GT1b and GM1. Besides, functions as receptor for chondroitin sulfate proteoglycans . Intracellular signaling cascades are triggered via the coreceptor NGFR. Signaling mediates activation of Rho and downstream reorganization of the actin cytoskeleton. Plays a role in regulating axon regeneration and neuronal plasticity in the adult central nervous system.
**SDK1**	12,35511	2,87E-12	Protein sidekick-1; Adhesion molecule that promotes lamina-specific synaptic connections in the retina. Expressed in specific subsets of interneurons and retinal ganglion cells (RGCs) and promotes synaptic connectivity via homophilic interactions.
**SEMA7A**	9,596869	1,58E-08	emaphorin-7A; Plays an important role in integrin-mediated signaling and functions both in regulating cell migration and immune responses. Promotes formation of focal adhesion complexes, activation of the protein kinase PTK2/FAK1 and subsequent phosphorylation of MAPK1 and MAPK3. Promotes production of proinflammatory cytokines by monocytes and macrophages. Plays an important role in modulating inflammation and T-cell-mediated immune responses. Promotes axon growth in the embryonic olfactory bulb. Promotes attachment, spreading and dendrite outgrowth in melanocytes.
**SH2B2**	7,492101	4,06E-15	H2B adapter protein 2; Adapter protein for several members of the tyrosine kinase receptor family. Involved in multiple signaling pathways.
**SOX8**	5,612484	0,000137	Transcription factor SOX-8; May play a role in central nervous system, limb and facial development. May be involved in male sex determination. Binds the consensus motif 5'-[AT][AT]CAA[AT]G-3'.

**SPINT1**	21,58251	1,26E-23	Kunitz-type protease inhibitor 1; Inhibitor of HGF activator. Also acts as an inhibitor of matriptase (ST14).
**SPRY3**	8,933214	1,9E-10	Protein sprouty homolog 3; May function as an antagonist of fibroblast growth factor (FGF) pathways and may negatively modulate respiratory organogenesis; Belongs to the sprouty family.
**TAL2**	7,622134	0,002611	T-cell acute lymphocytic leukemia protein 2; TAL bHLH transcription factor 2.
**TBX1**	21,00079	6E-18	T-box transcription factor TBX1; Probable transcriptional regulator involved in developmental processes.
**TENM2**	14,17268	7,06E-13	Ten-2 intracellular domain; Involved in neural development, regulating the establishment of proper connectivity within the nervous system. Promotes the formation of filopodia and enlarged growth cone in neuronal cells. Induces homophilic cell-cell adhesion.
**TENM4**	6,196145	1,13E-13	Teneurin-4; Involved in neural development, regulating the establishment of proper connectivity within the nervous system. Plays a role in the establishment of the anterior-posterior axis during gastrulation. Regulates the differentiation and cellular process formation of oligodendrocytes and myelination of small-diameter axons in the central nervous system (CNS). Promotes activation of focal adhesion kinase.
**TRPV4**	5,419075	3,76E-07	Transient receptor potential cation channel subfamily V member 4; Non-selective calcium permeant cation channel involved in osmotic sensitivity and mechanosensitivity. Activation by exposure to hypotonicity within the physiological range exhibits an outward rectification. Also activated by heat, low pH, citrate and phorbol esters. Increase of intracellular Ca(2+) potentiates currents.
**VEGFA**	11,94751	2,41E-24	Growth factor active in angiogenesis, vasculogenesis and endothelial cell growth. Induces endothelial cell proliferation, promotes cell migration, inhibits apoptosis and induces permeabilization of blood vessels. Binds to the NRP1/neuropilin-1 receptor. Binding to NRP1 initiates a signaling pathway needed for motor neuron axon guidance and cell body migration, including for the caudal migration of facial motor neurons from rhombomere 4 to rhombomere 6 during embryonic development.
**VEGFC**	9,867948	4,58E-15	Vascular endothelial growth factor C; Growth factor active in angiogenesis, and endothelial cell growth, stimulating their proliferation and migration and also has effects on the permeability of blood vessels. May function in angiogenesis of the venous and lymphatic vascular systems during embryogenesis, and also in the maintenance of differentiated lymphatic endothelium in adults.
**VLDLR**	22,84937	1,03E-49	Very low-density lipoprotein receptor; Binds VLDL and transports it into cells by endocytosis. In order to be internalized, the receptor-ligand complexes must first cluster into clathrin-coated pits.
**WNT11**	13,7125	1,23E-12	Protein Wnt-11; Ligand for members of the frizzled family of seven transmembrane receptors. Probable developmental protein. May be a signaling molecule which affects the development of discrete regions of tissues. Is likely to signal over only few cell diameters.
**WNT4**	15,35733	5,79E-08	Protein Wnt-4; Ligand for members of the frizzled family of seven transmembrane receptors

Supp. Table S3. Statistically up-regulated genes fc.COMB/CTR > 3, implicated in myeloid differentiation.

**Gene name**	**fc.COMB/CTR**	**bh.pvalue**	**Function**
**ARID2**	3,038133	1,27E-06	AT-rich interactive domain-containing protein 2; Involved in transcriptional activation and repression of select genes by chromatin remodeling (alteration of DNA-nucleosome topology). Required for the stability of the SWI/SNF chromatin remodeling complex SWI/SNF-B (PBAF). May be involved in targeting the complex to different genes.
**CARD11**	3,718161	0,016239	Caspase recruitment domain-containing protein 11; Involved in the costimulatory signal essential for T-cell receptor (TCR)-mediated T-cell activation. Its binding to DPP4 induces T-cell proliferation and NF-kappa-B activation in a T-cell receptor/CD3-dependent manner. Activates NF-kappa-B via BCL10 and IKK. Stimulates the phosphorylation of BCL10. Also activates the TORC1 signaling pathway.
**CD274**	6,373297	2,76E-05	Programmed cell death 1 ligand 1; Plays a critical role in induction and maintenance of immune tolerance to self. As a ligand for the inhibitory receptor PDCD1/PD-1, modulates the activation threshold of T-cells and limits T-cell effector response. Through a yet unknown activating receptor, may costimulate T-cell subsets that predominantly produce interleukin-10 (IL10).
**CDKN1A**	4,076187	1,74E-15	Cyclin-dependent kinase inhibitor 1; May be involved in p53/TP53 mediated inhibition of cellular proliferation in response to DNA damage.

**CEACAM1**	18,22363	3,38E-06	Carcinoembryonic antigen-related cell adhesion molecule 1; Cell adhesion protein that mediates homophilic cell adhesion in a calcium-independent manner. Plays a role as coinhibitory receptor in immune response, insulin action and functions also as an activator during angiogenesis. Its coinhibitory receptor function is phosphorylation- and PTPN6 -dependent, which in turn, suppress signal transduction of associated receptors by dephosphorylation of their downstream effectors. Plays a role in immune response, of T cells, natural killer (NK) and neutrophils.
**CEBPG**	3,5358	9,25E-13	CCAAT/enhancer-binding protein gamma; Transcription factor that binds to the promoter and the enhancer regions of target genes. Binds to the enhancer element PRE-I (positive regulatory element-I) of the IL-4 gene. Binds to the promoter and the enhancer of the immunoglobulin heavy chain. Binds to GPE1, a cis-acting element in the G-CSF gene promoter.
**CXCL8**	54,19723	1,07E-10	Interleukin-8; IL-8 is a chemotactic factor that attracts neutrophils, basophils, and T-cells, but not monocytes. It is also involved in neutrophil activation. It is released from several cell types in response to an inflammatory stimulus. IL-8(6-77) has a 5-10-fold higher activity on neutrophil activation, IL-8(5-77) has increased activity on neutrophil activation and IL-8(7-77) has a higher affinity to receptors CXCR1 and CXCR2 as compared to IL-8(1-77), respectively.
**DPP4**	3,27444	0,000291	Dipeptidyl peptidase 4 membrane form; Cell surface glycoprotein receptor involved in the costimulatory signal essential for T-cell receptor (TCR)-mediated T- cell activation. Acts as a positive regulator of T-cell coactivation, by binding at least ADA, CAV1, IGF2R, and PTPRC. Its binding to CAV1 and CARD11 induces T-cell proliferation and NF-kappa-B activation in a T-cell receptor/CD3-dependent manner.
**DYSF**	5,210954	2,04E-05	Dysferlin; Key calcium ion sensor involved in the Ca(2+)-triggered synaptic vesicle-plasma membrane fusion.
**EGR1**	3,136762	7,1E-07	Early growth response protein 1; Transcriptional regulator. Recognizes and binds to the DNA sequence 5'-GCG(T/G)GGGCG-3'(EGR-site) in the promoter region of target genes. Binds double-stranded target DNA, irrespective of the cytosine methylation status. Regulates the transcription of numerous target genes, and thereby plays an important role in regulating the response to growth factors, DNA damage, and ischemia. Plays a role in the regulation of cell survival, proliferation and cell death.
**F2RL1**	4,492837	2,89E-07	Proteinase-activated receptor 2, alternate cleaved 1; Receptor for trypsin and trypsin-like enzymes coupled to G proteins. Its function is mediated through the activation of several signaling pathways including phospholipase C (PLC), intracellular calcium, mitogen-activated protein kinase (MAPK), I-kappaB kinase/NF-kappaB and Rho.
**GPR137B**	7,528829	1,05E-30	Integral membrane protein GPR137B; Lysosomal integral membrane protein that regulates the localization and activity of mTORC1, a signaling complex promoting cell growth in response to growth factors, energy levels, and amino acids. Interacts with Rag GTPases and increases the lysosomial localization and activity of Rag GTPases and thereby regulates mTORC1 translocation and activity in lysosome.
**HLA-A**	4,01315	1,18E-14	HLA class I histocompatibility antigen, A alpha chain; Antigen-presenting major histocompatibility complex class I (MHCI) molecule. In complex with B2M/beta 2 microglobulin displays primarily viral and tumor-derived peptides on antigen-presenting cells for recognition by alpha-beta T cell receptor (TCR) on HLA-A-restricted CD8-positive T cells, guiding antigen-specific T cell immune response to eliminate infected or transformed cells.
**HLA-DPA1**	3,685418	8,93E-06	HLA class II histocompatibility antigen, DP alpha 1 chain; Binds peptides derived from antigens that access the endocytic route of antigen presenting cells (APC) and presents them on the cell surface for recognition by the CD4 T-cells. The peptide binding cleft accommodates peptides of 10-30 residues. The peptides presented by MHC class II molecules are generated mostly by degradation of proteins that access the endocytic route, where they are processed by lysosomal proteases and other hydrolases.
**HLA-DPB1**	3,968975	7,18E-07	HLA class II histocompatibility antigen, DP beta 1 chain; Binds peptides derived from antigens that access the endocytic route of antigen presenting cells (APC) and presents them on the cell surface for recognition by the CD4 T-cells. The peptide binding cleft accommodates peptides of 10-30 residues. The peptides presented by MHC class II molecules are generated mostly by degradation of proteins that access the endocytic route, where they are processed by lysosomal proteases and other hydrolases.
**HLA-G**	8,415614	2,38E-27	Soluble HLA class I histocompatibility antigen, alpha chain G; [Isoform 1]: Non-classical major histocompatibility class Ib molecule involved in immune regulatory processes at the maternal-fetal interface. In complex with B2M/beta-2 microglobulin binds a limited repertoire of nonamer self-peptides derived from intracellular proteins including histones and ribosomal proteins.
**IFNE**	3,173894	0,011679	Interferon epsilon; Type I interferon required for maintaining basal levels of IFN-regulated genes, including 2'-5'-oligoadenylate synthetase, IRF7 and ISG15.
**IL12A**	4,607256	2,9E-07	Interleukin-12 subunit alpha; Cytokine that can act as a growth factor for activated T and NK cells, enhance the lytic activity of NK/lymphokine-activated killer cells, and stimulate the production of IFN-gamma by resting PBMC.
**INHA**	6,567399	1,19E-08	Inhibin alpha chain; Inhibins and activins inhibit and activate, respectively, the secretion of follitropin by the pituitary gland. Inhibins/activins are involved in regulating a number of diverse functions such as hypothalamic and pituitary hormone secretion, gonadal hormone secretion, germ cell development and maturation, erythroid differentiation, insulin secretion, nerve cell survival, embryonic axial development or bone growth, depending on their subunit composition.
**IRF4**	3,90616	0,001097	Interferon regulatory factor 4; Transcriptional activator. Binds to the interferon-stimulated response element (ISRE) of the MHC class I promoter. Binds the immunoglobulin lambda light chain enhancer, together with PU.1. Probably plays a role in ISRE-targeted signal transduction mechanisms specific to lymphoid cells. Involved in CD8(+) dendritic cell differentiation
**JMJD6**	3,227592	6,48E-11	Bifunctional arginine demethylase and lysyl-hydroxylase JMJD6; Dioxygenase that can both act as a arginine demethylase and a lysyl-hydroxylase. Acts as a lysyl-hydroxylase that catalyzes 5-hydroxylation on specific lysine residues of target proteins such as U2AF2/U2AF65 and LUC7L2. Regulates RNA splicing by mediating 5-hydroxylation of U2AF2/U2AF65, affecting the pre-mRNA splicing activity of U2AF2/U2AF65. Hydroxylates its own N-terminus, which is required for homooligomerization.
**JUNB**	3,63835	1,07E-12	Transcription factor jun-B; Transcription factor involved in regulating gene activity following the primary growth factor response. Binds to the DNA sequence 5'-TGA[CG]TCA-3'; Belongs to the bZIP family. Jun subfamily.
**LFNG**	3,776286	0,00938	eta-1,3-N-acetylglucosaminyltransferase lunatic fringe; Glycosyltransferase that initiates the elongation of O-linked fucose residues attached to EGF-like repeats in the extracellular domain of Notch molecules.

**MFHAS1**	11,31433	1,33E-07	Malignant fibrous histiocytoma-amplified sequence 1; Probable GTP-binding protein. Functions in innate immunity and more specifically the inflammatory response as a regulator of the Toll-like receptor TLR2 and TLR4 signaling pathways. Negatively regulates the part of the TLR4 signaling pathway that leads to the activation of the transcription factor AP-1. By retaining the phosphatase complex PP2A into the cytoplasm, prevents the dephosphorylation of the AP-1 subunit JUN which is required for proper activation of the transcription factor.
**MFNG**	13,65442	1,06E-11	Beta-1,3-N-acetylglucosaminyltransferase manic fringe; Glycosyltransferase that initiates the elongation of O-linked fucose residues attached to EGF-like repeats in the extracellular domain of Notch molecules. Modulates NOTCH1 activity by modifying O-fucose residues at specific EGF-like domains resulting in inhibition of NOTCH1 activation by JAG1 and enhancement of NOTCH1 activation by DLL1 via an increase in its binding to DLL1.
**MT1G**	48,19312	1,56E-29	Metallothionein-1G; Metallothioneins have a high content of cysteine residues that bind various heavy metals; these proteins are transcriptionally regulated by both heavy metals and glucocorticoids.
**NFIL3**	3,936429	7,6E-12	Nuclear factor interleukin-3-regulated protein; Acts as a transcriptional regulator that recognizes and binds to the sequence 5'-[GA]TTA[CT]GTAA[CT]-3', a sequence present in many cellular and viral promoters. Represses transcription from promoters with activating transcription factor (ATF) sites. Represses promoter activity in osteoblasts. Represses transcriptional activity of PER1. Represses transcriptional activity of PER2 via the B-site on the promoter. Activates transcription from the interleukin-3 promoter in T-cells.
**NFKBID**	3,953235	3,42E-06	NF-kappa-B inhibitor delta; Regulates the expression of IL-2, IL-6, and other cytokines through regulation on NF-kappa-B activity. Functions in the regulation of inflammatory responses. Involved in the induction of T helper 17 cells (Th17) differentiation upon recognition of antigen by T cell antigen receptor (TCR). May also regulate TCR-induced negative selection of thymocytes.
**NFKBIZ**	3,053244	2,07E-08	NF-kappa-B inhibitor zeta; Involved in regulation of NF-kappa-B transcription factor complexes. Inhibits NF-kappa-B activity without affecting its nuclear translocation upon stimulation. Inhibits DNA-binding of RELA and NFKB1/p50, and of the NF-kappa-B p65-p50 heterodimer and the NF-kappa-B p50-p50 homodimer. Seems also to activate NF-kappa-B-mediated transcription. In vitro, upon association with NFKB1/p50 has transcriptional activation activity and, together with NFKB1/p50 and RELA, is recruited to LCN2 promoters.
**NR4A3**	11,41068	5,11E-08	Nuclear receptor subfamily 4 group A member 3; Transcriptional activator plays a role in the regulation of proliferation, survival and differentiation of many different cell types and also in metabolism and inflammation.
**PAG1**	6,405957	7,15E-15	Phosphoprotein associated with glycosphingolipid-enriched microdomains 1; Negatively regulates TCR (T-cell antigen receptor)-mediated signaling in T-cells and FCER1 (high affinity immunoglobulin epsilon receptor)-mediated signaling in mast cells. Promotes CSK activation and recruitment to lipid rafts, which results in LCK inhibition. Inhibits immunological synapse formation by preventing dynamic arrangement of lipid raft proteins. May be involved in cell adhesion signaling.
**PCK1**	16,17959	8,27E-09	Phosphoenolpyruvate carboxykinase, cytosolic [GTP]; Regulates cataplerosis and anaplerosis, the processes that control the levels of metabolic intermediates in the citric acid cycle. At low glucose levels, it catalyzes the cataplerotic conversion of oxaloacetate (OAA) to phosphoenolpyruvate (PEP), the rate-limiting step in the metabolic pathway that produces glucose from lactate and other precursors derived from the citric acid cycle. At high glucose levels, it catalyzes the anaplerotic conversion of phosphoenolpyruvate to oxaloacetate.
**PRR7**	9,57425	6,24E-27	Proline-rich protein 7; Acts as a synapse-to-nucleus messenger to promote NMDA receptor-mediated excitotoxicity in neurons in a JUN-dependent manner. Inhibits ubiquitination-mediated degradation and promotes phosphorylation and transcriptional activity of transcription factor JUN. Might play a redundant role in the regulation of T cell receptor signaling. Might promote apoptosis in T cells.
**RAG1**	3,701124	0,029441	V(D)J recombination-activating protein 1; Catalytic component of the RAG complex, a multiprotein complex that mediates the DNA cleavage phase during V(D)J recombination. V(D)J recombination assembles a diverse repertoire of immunoglobulin and T-cell receptor genes in developing B and T- lymphocytes through rearrangement of different V (variable), in some cases D (diversity), and J (joining) gene segments.
**RASAL3**	25,45928	1,99E-25	RAS protein activator like-3; Functions as a Ras GTPase-activating protein. Plays an important role in the expansion and functions of natural killer T (NKT) cells in the liver by negatively regulating RAS activity and the down- stream ERK signaling pathway.
**RBPJ**	3,710435	2,84E-13	Recombining binding protein suppressor of hairless; Transcriptional regulator that plays a central role in Notch signaling, a signaling pathway involved in cell-cell communication that regulates a broad spectrum of cell-fate determinations. Acts as a transcriptional repressor when it is not associated with Notch proteins. When associated with some NICD product of Notch proteins (Notch intracellular domain), it acts as a transcriptional activator that activates transcription of Notch target genes.
**SELPLG**	7,076405	4,43E-10	P-selectin glycoprotein ligand 1; A SLe(x)-type proteoglycan, which through high affinity, calcium-dependent interactions with E-, P- and L-selectins, mediates rapid rolling of leukocytes over vascular surfaces during the initial steps in inflammation. Critical for the initial leukocyte capture.
**SLC11A1**	3,836155	0,01353	Natural resistance-associated macrophage protein 1; Divalent transition metal (iron and manganese) transporter involved in iron metabolism and host resistance to certain pathogens. Macrophage-specific membrane transport function.
**SLC7A1**	3,150323	2,74E-07	High affinity cationic amino acid transporter 1; High-affinity, low capacity permease involved in the transport of the cationic amino acids (arginine, lysine and ornithine) in non-hepatic tissues.
**SOCS1**	7,357771	0,000132	Suppressor of cytokine signaling 1; SOCS family proteins form part of a classical negative feedback system that regulates cytokine signal transduction. SOCS1 is involved in negative regulation of cytokines that signal through the JAK/STAT3 pathway. Through binding to JAKs, inhibits their kinase activity. In vitro, also suppresses Tec protein-tyrosine activity. Appears to be a major regulator of signaling by interleukin 6 (IL6) and leukemia inhibitory factor (LIF). Regulates interferon-gamma mediated sensory neuron survival.
**TFRC**	4,382754	2,23E-15	Transferrin receptor protein 1, serum form; Cellular uptake of iron occurs via receptor-mediated endocytosis of ligand-occupied transferrin receptor into specialized endosomes. Endosomal acidification leads to iron release. The apotransferrin-receptor complex is then recycled to the cell surface with a return to neutral pH and the concomitant loss of affinity of apotransferrin for its receptor. Transferrin receptor is necessary for development of erythrocytes and the nervous system.
**THEMIS2**	3,550432	0,001358	May constitute a control point in macrophage inflammatory response, promoting LPS-induced TNF production.

**TNFRSF13C**	6,645808	0,000114	Tumor necrosis factor receptor superfamily member 13C; B-cell receptor specific for TNFSF13B/TALL1/BAFF/BLyS. Promotes the survival of mature B-cells and the B-cell response.
**TNFSF13B**	3,889675	3,17E-07	Tumor necrosis factor ligand superfamily member 13b, membrane form; Cytokine that binds to TNFRSF13B/TACI and TNFRSF17/BCMA. TNFSF13/APRIL binds to the same 2 receptors. Together, they form a 2 ligands -2 receptors pathway involved in the stimulation of B- and T- cell function and the regulation of humoral immunity. A third B-cell specific BAFF-receptor (BAFFR/BR3) promotes the survival of mature B- cells and the B-cell response.
**TNFSF9**	4,749556	9,98E-07	Tumor necrosis factor ligand superfamily member 9; Cytokine that binds to TNFRSF9. Induces the proliferation of activated peripheral blood T-cells. May have a role in activation- induced cell death (AICD). May play a role in cognate interactions between T-cells and B-cells/macrophages; Belongs to the tumor necrosis factor family.
**TPD52**	8,864758	2,63E-10	Tumor protein D52; Belongs to the TPD52 family.
**ULBP2**	7,77324	4,33E-08	UL16-binding protein 2; Binds and activates the KLRK1/NKG2D receptor, mediating natural killer cell cytotoxicity.
**UNC13D**	3,390745	0,02303	Protein unc-13 homolog D; Plays a role in cytotoxic granule exocytosis in lymphocytes. Required for both granule maturation and granule docking and priming at the immunologic synapse. Regulates assembly of recycling and late endosomal structures, leading to the formation of an endosomal exocytic compartment that fuses with perforin-containing granules at the immunologic synapse and licences them for exocytosis. Regulates Ca(2+)- dependent secretory lysosome exocytosis in mast cells.
**WNK4**	3,534278	9,5E-05	Serine/threonine-protein kinase WNK4; Serine/threonine kinase which plays an important role in the regulation of electrolyte homeostasis, cell signaling, survival and proliferation. Acts as an activator and inhibitor of sodium-coupled chloride cotransporters and potassium-coupled chloride cotransporters respectively. Activates SCNN1A, SCNN1B, SCNN1D, SGK1, TRPV5 and TRPV6. Regulates the activity of the thiazide-sensitive Na-Cl cotransporter, SLC12A3, by phosphorylation which appears to prevent membrane trafficking of SLC12A3.
**ZBTB1**	3,587333	5,24E-12	Zinc finger and BTB domain-containing protein 1; Acts as a transcriptional repressor. Represses cAMP-responsive element (CRE)-mediated transcriptional activation. In addition, has a role in translesion DNA synthesis. Requires for UV-inducible RAD18 loading, PCNA monoubiquitination, POLH recruitment to replication factories and efficient translesion DNA synthesis. Plays a key role in the transcriptional regulation of T lymphocyte development.

Supp. Table S4. Statistically down-regulated genes fc.COMB/CTR < -2.

**Gene name**	**fc.COMB/CTR**	**bh.pvalue**	**Description**
**ABCC6**	-2,19611	0,024193	Multidrug resistance-associated protein 6; [Isoform 1]: May participate directly in the active transport of drugs into subcellular organelles or influence drug distribution indirectly. Transports glutathione conjugates as leukotriene-c4 (LTC4) and N-ethylmaleimide S-glutathione (NEM-GS). Belongs to the ABC transporter superfamily. ABCC family. Conjugate transporter (TC 3.A.1.208) subfamily.
**ACAD9**	-2,00773	0,000195	Complex I assembly factor ACAD9, mitochondrial; As part of the MCIA complex, primarily participates to the assembly of the mitochondrial complex I and therefore plays a role in oxidative phosphorylation. This moonlighting protein has also a dehydrogenase activity toward a broad range of substrates with greater specificity for long-chain unsaturated acyl-CoAs. However, in vivo, it does not seem to play a primary role in fatty acid oxidation. In addition, the function in complex I assembly is independent of the dehydrogenase activity of the protein.
**ACSF2**	-3,388010	2,19261E-11	Medium-chain acyl-CoA ligase ACSF2, mitochondrial; Acyl-CoA synthases catalyze the initial reaction in fatty acid metabolism, by forming a thioester with CoA. Has some preference toward medium-chain substrates.
**ADCK1**	-2,4418	8,21E-05	AarF domain-containing protein kinase 1; Appears to be essential for maintaining mitochondrial cristae formation and mitochondrial function by acting via YME1L1 in a kinase- independent manner to regulate essential mitochondrial structural proteins OPA1 and IMMT.
**AIFM1**	-2,48215	1,54E-06	Apoptosis-inducing factor 1, mitochondrial; Functions both as NADH oxidoreductase and as regulator of apoptosis. In response to apoptotic stimuli, it is released from the mitochondrion intermembrane space into the cytosol and to the nucleus, where it functions as a proapoptotic factor in a caspase-independent pathway. The soluble form (AIFsol) found in the nucleus induces 'parthanatos' i.e. caspase-independent fragmentation of chromosomal DNA.
**ATAD3A**	-2,17148	0,000441	ATPase family AAA domain-containing protein 3A; Essential for mitochondrial network organization, mitochondrial metabolism and cell growth at organism and cellular level. May play an important role in mitochondrial protein synthesis. May also participate in mitochondrial DNA replication. May bind to mitochondrial DNA D-loops and contribute to nucleoid stability. Required for enhanced channeling of cholesterol for hormone-dependent steroidogenesis.
**ATP23**	-2,57462	0,012559	Mitochondrial inner membrane protease ATP23 homolog; ATP23 metallopeptidase and ATP synthase assembly factor homolog.
**ATP5F1A**	-2,05636	8,48E-05	ATP synthase subunit alpha, mitochondrial.
**ATP5F1E**	-2,10579	0,000202	ATP synthase subunit epsilon, mitochondrial.
**ATP5MC1**	-4,38935	1,12E-14	ATP synthase F(0) complex subunit C1, mitochondrial.
**ATP5ME**	-2,23545	0,000136	ATP synthase subunit e, mitochondrial, N-terminally processed.
**ATP5PD**	-2,05443	0,000286	ATP synthase subunit d, mitochondrial.
**AURKA**	-2,18455	0,010664	Aurora kinase A; Mitotic serine/threonine kinase that contributes to the regulation of cell cycle progression. Associates with the centrosome and the spindle microtubules during mitosis and plays a critical role in various

			mitotic events including the establishment of mitotic spindle, centrosome duplication, centrosome separation as well as maturation, chromosomal alignment, spindle assembly checkpoint, and cytokinesis.
**BCS1L**	-2,6334	2,46E-07	Mitochondrial chaperone BCS1; Chaperone necessary for the assembly of mitochondrial respiratory chain complex III. Plays an important role in the maintenance of mitochondrial tubular networks, respiratory chain assembly and formation of the LETM1 complex.
**CAMKMT**	-3,80925	4,97E-09	Calmodulin-lysine N-methyltransferase; Catalyzes the trimethylation of 'Lys-116' in calmodulin. Belongs to the class I-like SAM-binding methyltransferase superfamily. CLNMT methyltransferase family.
**CCNB1**	-3,1844	1,81E-08	G2/mitotic-specific cyclin-B1; Essential for the control of the cell cycle at the G2/M (mitosis) transition; Belongs to the cyclin family. Cyclin AB subfamily.
**CDKN2A**	-2,00806	0,000296	Cyclin-dependent kinase inhibitor 2A; Acts as a negative regulator of the proliferation of normal cells by interacting strongly with CDK4 and CDK6. This inhibits their ability to interact with cyclins D and to phosphorylate the retinoblastoma protein; Belongs to the CDKN2 cyclin-dependent kinase inhibitor family.
**CHCHD10**	-6,89295	9,09E-06	Coiled-coil-helix-coiled-coil-helix domain-containing protein 10, mitochondrial; May be involved in the maintenance of mitochondrial organization and mitochondrial cristae structure.
**CHCHD3**	-2,01374	0,000169	MICOS complex subunit MIC19; Component of the MICOS complex, a large protein complex of the mitochondrial inner membrane that plays crucial roles in the maintenance of crista junctions, inner membrane architecture, and formation of contact sites to the outer membrane. Has also been shown to function as a transcription factor which binds to the BAG1 promoter and represses BAG1 transcription. Plays an important role in the maintenance of the MICOS complex stability and the mitochondrial cristae morphology.
**CHCHD4**	-2,33203	0,000129	Mitochondrial intermembrane space import and assembly protein 40; Central component of a redox-sensitive mitochondrial intermembrane space import machinery which is required for the biogenesis of respiratory chain complexes. Functions as chaperone and catalyzes the formation of disulfide bonds in substrate proteins, such as COX17, COX19, MICU1 and COA7. Required for the import and folding of small cysteine-containing proteins (small Tim) in the mitochondrial intermembrane space (IMS). Required for the import of COA7 in the IMS.
**CIBAR1**	-2,82957	1,37E-06	FAM92A - Protein FAM92A; Acts as a positive regulator of ciliary hedgehog signaling. Probable regulator of ciliogenesis involved in limb morphogenesis. In cooperation with CBY1 it is involved in the recruitment and fusion of endosomal vesicles at distal appendages during early stages of ciliogenesis; Belongs to the FAM92 family.
**COA6**	-3,16994	9,04E-06	Cytochrome c oxidase assembly factor 6 homolog; Involved in the maturation of the mitochondrial respiratory chain complex IV subunit MT-CO2/COX2. Thereby, may regulate early steps of complex IV assembly. Mitochondrial respiratory chain complex IV or cytochrome c oxidase is the component of the respiratory chain that catalyzes the transfer of electrons from intermembrane space cytochrome c to molecular oxygen in the matrix and as a consequence contributes to the proton gradient involved in mitochondrial ATP synthesis.
**COX16**	-2,24108	4,87E-05	Cytochrome c oxidase assembly protein COX16 homolog, mitochondrial; Required for the assembly of the mitochondrial respiratory chain complex IV (CIV), also known as cytochrome c oxidase. Promotes the insertion of copper into the active site of cytochrome c oxidase subunit II (MT-CO2/COX2). Interacts specifically with newly synthesized MT-CO2/COX and its copper center-forming metallochaperones SCO1, SCO2 and COA6.
**COX7A1**	-2,01283	0,000137	Cytochrome c oxidase subunit 7A1, mitochondrial; Component of the cytochrome c oxidase, the last enzyme in the mitochondrial electron transport chain which drives oxidative phosphorylation. The respiratory chain contains 3 multisubunit complexes succinate dehydrogenase (complex II, CII), ubiquinol- cytochrome c oxidoreductase (cytochrome b-c1 complex, complex III, CIII) and cytochrome c oxidase (complex IV, CIV), that cooperate to transfer electrons derived from NADH and succinate to molecular oxygen, creating an electrochemical gradient over the inner membrane.
**CYB561**	-2,4155	2,56E-05	Cytochrome b561; Secretory vesicle-specific electron transport protein.
**CYB5A**	-2,04534	0,000319	Cytochrome b5; Cytochrome b5 is a membrane-bound hemoprotein functioning as an electron carrier for several membrane-bound oxygenases.
**DARS2**	-2,2979	0,000646	Aspartate--tRNA ligase, mitochondrial; aspartyl-tRNA synthetase 2, mitochondrial.
**DNAJC15**	-3,92796	1,3E-09	DnaJ homolog subfamily C member 15; Negative regulator of the mitochondrial respiratory chain. Prevents mitochondrial hyperpolarization state and restricts mitochondrial generation of ATP. Acts as an import component of the TIM23 translocase complex. Stimulates the ATPase activity of HSPA9.
**DNAJC19**	-2,36655	3,37E-05	Mitochondrial import inner membrane translocase subunit TIM14; Probable component of the PAM complex, a complex required for the translocation of transit peptide-containing proteins from the inner membrane into the mitochondrial matrix in an ATP-dependent manner. May act as a co-chaperone that stimulate the ATP-dependent activity.
**ELAC2**	-2,50945	2,46E-07	Zinc phosphodiesterase ELAC protein 2; Zinc phosphodiesterase, which displays mitochondrial tRNA 3'-processing endonuclease activity. Involved in tRNA maturation, by removing a 3'-trailer from precursor tRNA.
**EPM2A**	-2,29433	0,005397	Laforin; Plays an important role in preventing glycogen hyperphosphorylation and the formation of insoluble aggregates, via its activity as glycogen phosphatase, and by promoting the ubiquitination of proteins involved in glycogen metabolism via its interaction with the E3 ubiquitin ligase NHLRC1/malin.
**ETFA**	-2,08466	0,000121	Electron transfer flavoprotein subunit alpha, mitochondrial; Heterodimeric electron transfer flavoprotein that accepts electrons from several mitochondrial dehydrogenases, including acyl-CoA dehydrogenases, glutaryl-CoA and sarcosine dehydrogenase. It transfers the electrons to the main mitochondrial respiratory chain via ETF-ubiquinone oxidoreductase (ETF dehydrogenase). Required for normal mitochondrial fatty acid oxidation and normal amino acid metabolism.
**ETFB**	-2,33505	0,000124	Electron transfer flavoprotein subunit beta; Heterodimeric electron transfer flavoprotein that accepts electrons from several mitochondrial dehydrogenases, including acyl-CoA dehydrogenases, glutaryl-CoA and sarcosine dehydrogenase. Required for normal mitochondrial fatty acid oxidation and normal amino acid metabolism. ETFB binds an AMP molecule that probably has a purely structural role.
**ETFRF1**	-2,76523	0,001012	Electron transfer flavoprotein regulatory factor 1; Acts as a regulator of the electron transfer flavoprotein by promoting the removal of flavin from the ETF holoenzyme (composed of ETFA and ETFB); Belongs to the complex I LYR family.

**FASTKD2**	-2,09241	0,001124	FAST kinase domain-containing protein 2, mitochondrial; Plays an important role in assembly of the mitochondrial large ribosomal subunit. As a component of a functional protein-RNA module, consisting of RCC1L, NGRN, RPUSD3, RPUSD4, TRUB2, FASTKD2 and 16S mitochondrial ribosomal RNA (16S mt-rRNA),
**FDX1**	-2,17458	0,002234	Adrenodoxin, mitochondrial; Essential for the synthesis of various steroid hormones. Participates in the reduction of mitochondrial cytochrome P450 for steroidogenesis.
**FDX2**	-2,28625	0,000153	Ferredoxin-2, mitochondrial; Essential for heme A and Fe/S protein biosynthesis.
**FMC1**	-2,13325	0,002174	Plays a role in the assembly/stability of the mitochondrial membrane ATP synthase (F(1)F(0) ATP synthase or Complex V) ; Belongs to the FMC1 family.
**FUNDC1**	-2,12428	0,000596	FUN14 domain-containing protein 1; Acts as an activator of hypoxia-induced mitophagy, an important mechanism for mitochondrial quality control.
**GFUS**	-2,51724	8,51E-07	GDP-L-fucose synthase; Catalyzes the two-step NADP-dependent conversion of GDP-4- dehydro-6-deoxy-D-mannose to GDP-fucose, involving an epimerase and a reductase reaction.
**GGCT**	-3,78765	1,47E-10	Gamma-glutamylcyclotransferase; Catalyzes the formation of 5-oxoproline from gamma-glutamyl dipeptides and may play a significant role in glutathione homeostasis. Induces release of cytochrome c from mitochondria with resultant induction of apoptosis; Belongs to the gamma-glutamylcyclotransferase family.
**HGF**	-3,53344	0,001055	Hepatocyte growth factor alpha chain; Potent mitogen for mature parenchymal hepatocyte cells, seems to be a hepatotrophic factor, and acts as a growth factor for a broad spectrum of tissues and cell types. Activating ligand for the receptor tyrosine kinase MET by binding to it and promoting its dimerization.
**HSD17B10**	-3,36708	2,5E-11	3-hydroxyacyl-CoA dehydrogenase type-2; Mitochondrial dehydrogenase that catalyzes the beta-oxidation at position 17 of androgens and estrogens and has 3-alpha- hydroxysteroid dehydrogenase activity with androsterone. Catalyzes the third step in the beta-oxidation of fatty acids.
**HSPD1**	-2,50127	6,42E-06	60 kDa heat shock protein, mitochondrial; Chaperonin implicated in mitochondrial protein import and macromolecular assembly. Together with Hsp10, facilitates the correct folding of imported proteins. May also prevent misfolding and promote the refolding and proper assembly of unfolded polypeptides generated under stress conditions in the mitochondrial matrix.
**IGF1**	-4,33709	0,000519	Insulin-like growth factor I; The insulin-like growth factors, isolated from plasma, are structurally and functionally related to insulin but have a much higher growth-promoting activity.
**IMMP1L**	-3,01918	0,006947	Mitochondrial inner membrane protease subunit 1; Catalyzes the removal of transit peptides required for the targeting of proteins from the mitochondrial matrix, across the inner membrane, into the inter-membrane space. Known to process the nuclear encoded protein DIABLO.
**MAIP1**	-2,19638	0,000812	m-AAA protease-interacting protein 1, mitochondrial; Promotes sorting of SMDT1/EMRE in mitochondria by ensuring its maturation. Interacts with the transit peptide region of SMDT1/EMRE precursor protein in the mitochondrial matrix, leading to protect it against protein degradation by YME1L1, thereby ensuring SMDT1/EMRE maturation by the mitochondrial processing peptidase (PMPCA and PMPCB).
**MAP1LC3 C**	-5,3434	0,001508	Microtubule-associated proteins 1A/1B light chain 3C; Ubiquitin-like modifier that plays a crucial role in antibacterial autophagy (xenophagy) through the selective binding of CALCOCO2. Recruits all ATG8 family members to infecting bacteria such as S.Typhimurium. May also play a role in aggrephagy, the macroautophagic degradation of ubiquitinated and aggregated proteins.
**MIPEP**	-2,63324	4,58E-06	Mitochondrial intermediate peptidase; Cleaves proteins, imported into the mitochondrion, to their mature size.
**MSH2**	-2,29192	0,000869	DNA mismatch repair protein Msh2; Component of the post-replicative DNA mismatch repair system (MMR). Forms two different heterodimers: MutS alpha (MSH2-MSH6 heterodimer) and MutS beta (MSH2-MSH3 heterodimer) which binds to DNA mismatches thereby initiating DNA repair. When bound, heterodimers bend the DNA helix and shields approximately 20 base pairs. MutS alpha recognizes single base mismatches and dinucleotide insertion-deletion loops (IDL) in the DNA.
**MSTO1**	-2,65515	8,58E-08	Protein misato homolog 1; Involved in the regulation of mitochondrial distribution and morphology. Required for mitochondrial fusion and mitochondrial network formation.
**MYBBP1A**	-3,06824	3,97E-07	Myb-binding protein 1A; May activate or repress transcription via interactions with sequence specific DNA-binding proteins. Repression may be mediated at least in part by histone deacetylase activity (HDAC activity). Acts as a corepressor and in concert with CRY1, represses the transcription of the core circadian clock component PER2. Preferentially binds to dimethylated histone H3 'Lys-9' (H3K9me2) on the PER2 promoter.
**NDUFA12**	-3,21647	9,71E-08	NADH dehydrogenase [ubiquinone] 1 alpha subcomplex subunit 12; Accessory subunit of the mitochondrial membrane respiratory chain NADH dehydrogenase (Complex I), that is believed not to be involved in catalysis. Complex I functions in the transfer of electrons from NADH to the respiratory chain. The immediate electron acceptor for the enzyme is believed to be ubiquinone.
**NDUFA7**	-2,18246	7,06E-05	NADH dehydrogenase [ubiquinone] 1 alpha subcomplex subunit 7; Accessory subunit of the mitochondrial membrane respiratory chain NADH dehydrogenase (Complex I), that is believed not to be involved in catalysis. Complex I functions in the transfer of electrons from NADH to the respiratory chain. The immediate electron acceptor for the enzyme is believed to be ubiquinone.
**NDUFA9**	-2,04261	0,000195	NDUFA9 - NADH dehydrogenase [ubiquinone] 1 alpha subcomplex subunit 9, mitochondrial; Accessory subunit of the mitochondrial membrane respiratory chain NADH dehydrogenase (Complex I), that is believed not to be involved in catalysis. Required for proper complex I assembly. Complex I functions in the transfer of electrons from NADH to the respiratory chain. The immediate electron acceptor for the enzyme is believed to be ubiquinone.
**NDUFAF2**	-4,82889	1,07E-09	NADH dehydrogenase [ubiquinone] 1 alpha subcomplex assembly factor 2; Acts as a molecular chaperone for mitochondrial complex I assembly. Complex I functions in the transfer of electrons from NADH to the respiratory chain. The immediate electron acceptor for the enzyme is believed to be ubiquinone.

**NDUFAF4**	-4,51127	2,19E-11	NADH dehydrogenase [ubiquinone] 1 alpha subcomplex assembly factor 4; Involved in the assembly of mitochondrial NADH:ubiquinone oxidoreductase complex (complex I). May be involved in cell proliferation and survival of hormone-dependent tumor cells. May be a regulator of breast tumor cell invasion.
**NDUFAF5**	-2,24142	0,001051	Arginine-hydroxylase NDUFAF5, mitochondrial; Arginine hydroxylase involved in the assembly of mitochondrial NADH:ubiquinone oxidoreductase complex (complex I, MT- ND1) at early stages. Acts by mediating hydroxylation of 'Arg-111' of NDUFS7.
**NDUFB1**	-2,94007	9,61E-08	NADH dehydrogenase [ubiquinone] 1 beta subcomplex subunit 1; Accessory subunit of the mitochondrial membrane respiratory chain NADH dehydrogenase (Complex I) that is believed not to be involved in catalysis. Complex I functions in the transfer of electrons from NADH to the respiratory chain. The immediate electron acceptor for the enzyme is believed to be ubiquinone.
**NDUFB10**	-2,10048	0,000161	NADH dehydrogenase [ubiquinone] 1 beta subcomplex subunit 10; Accessory subunit of the mitochondrial membrane respiratory chain NADH dehydrogenase (Complex I), that is believed not to be involved in catalysis. Complex I functions in the transfer of electrons from NADH to the respiratory chain. The immediate electron acceptor for the enzyme is believed to be ubiquinone.
**NDUFB3**	-2,26972	6,43E-05	NADH dehydrogenase [ubiquinone] 1 beta subcomplex subunit 3; Accessory subunit of the mitochondrial membrane respiratory chain NADH dehydrogenase (Complex I), that is believed not to be involved in catalysis. Complex I functions in the transfer of electrons from NADH to the respiratory chain. The immediate electron acceptor for the enzyme is believed to be ubiquinone.
**NDUFB5**	-2,42285	9,27E-06	NADH dehydrogenase [ubiquinone] 1 beta subcomplex subunit 5, mitochondrial; Accessory subunit of the mitochondrial membrane respiratory chain NADH dehydrogenase (Complex I), that is believed not to be involved in catalysis. Complex I functions in the transfer of electrons from NADH to the respiratory chain. The immediate electron acceptor for the enzyme is believed to be ubiquinone.
**NDUFB6**	-3,01484	7,67E-08	NADH dehydrogenase [ubiquinone] 1 beta subcomplex subunit 6; Accessory subunit of the mitochondrial membrane respiratory chain NADH dehydrogenase (Complex I), that is believed not to be involved in catalysis. Complex I functions in the transfer of electrons from NADH to the respiratory chain. The immediate electron acceptor for the enzyme is believed to be ubiquinone.
**NDUFS2**	-2,08999	4,83E-05	NADH dehydrogenase [ubiquinone] iron-sulfur protein 2, mitochondrial; Core subunit of the mitochondrial membrane respiratory chain NADH dehydrogenase (Complex I) that is believed to belong to the minimal assembly required for catalysis. Complex I functions in the transfer of electrons from NADH to the respiratory chain. The immediate electron acceptor for the enzyme is believed to be ubiquinone.
**NDUFS3**	-2,30659	1,74E-05	NADH dehydrogenase [ubiquinone] iron-sulfur protein 3, mitochondrial; Core subunit of the mitochondrial membrane respiratory chain NADH dehydrogenase (Complex I) that is believed to belong to the minimal assembly required for catalysis. Complex I functions in the transfer of electrons from NADH to the respiratory chain. The immediate electron acceptor for the enzyme is believed to be ubiquinone.
**NDUFS5**	-2,08862	0,000198	NADH dehydrogenase [ubiquinone] iron-sulfur protein 5; Accessory subunit of the mitochondrial membrane respiratory chain NADH dehydrogenase (Complex I), that is believed not to be involved in catalysis. Complex I functions in the transfer of electrons from NADH to the respiratory chain. The immediate electron acceptor for the enzyme is believed to be ubiquinone.
**NDUFS6**	-2,73603	5,75E-08	NADH dehydrogenase [ubiquinone] iron-sulfur protein 6, mitochondrial; Accessory subunit of the mitochondrial membrane respiratory chain NADH dehydrogenase (Complex I), that is believed not to be involved in catalysis. Complex I functions in the transfer of electrons from NADH to the respiratory chain. The immediate electron acceptor for the enzyme is believed to be ubiquinone.
**NDUFS8**	-2,5279	6,63E-07	NADH dehydrogenase [ubiquinone] iron-sulfur protein 8, mitochondrial; Core subunit of the mitochondrial membrane respiratory chain NADH dehydrogenase (Complex I) that is believed to belong to the minimal assembly required for catalysis. Complex I functions in the transfer of electrons from NADH to the respiratory chain. The immediate electron acceptor for the enzyme is believed to be ubiquinone. May donate electrons to ubiquinone.
**NQO2**	-2,62867	6,14E-07	Ribosyldihydronicotinamide dehydrogenase [quinone]; The enzyme apparently serves as a quinone reductase in connection with conjugation reactions of hydroquinones involved in detoxification pathways as well as in biosynthetic processes such as the vitamin K-dependent gamma-carboxylation of glutamate residues in prothrombin synthesis; Belongs to the NAD(P)H dehydrogenase (quinone) family.
**OMA1**	-2,3375	0,000305	Metalloendopeptidase OMA1, mitochondrial; Metalloprotease that is part of the quality control system in the inner membrane of mitochondria. Following stress conditions that induce loss of mitochondrial membrane potential, mediates cleavage of OPA1 at S1 position, leading to OPA1 inactivation and negative regulation of mitochondrial fusion. May also cleave UQCC3 under these conditions. Its role in mitochondrial quality control is essential for regulating lipid metabolism as well as to maintain body temperature and energy expenditure under cold-stress conditions.
**OXA1L**	-2,261	1,33E-05	Mitochondrial inner membrane protein OXA1L; Required for the insertion of integral membrane proteins into the mitochondrial inner membrane. Essential for the activity and assembly of cytochrome oxidase. Required for the correct biogenesis of ATP synthase and complex I in mitochondria.
**PAM16**	-2,37	0,000242	Mitochondrial import inner membrane translocase subunit TIM16; Regulates ATP-dependent protein translocation into the mitochondrial matrix. Inhibits DNAJC19 stimulation of HSPA9/Mortalin ATPase activity; Belongs to the TIM16/PAM16 family.
**PARP1**	-2,31045	3,75E-06	Poly [ADP-ribose] polymerase 1; Poly-ADP-ribosyltransferase that mediates poly-ADP- ribosylation of proteins and plays a key role in DNA repair. Mainly mediates glutamate and aspartate ADP-ribosylation of target proteins: the ADP-D- ribosyl group of NAD(+) is transferred to the acceptor carboxyl group of glutamate and aspartate residues and further ADP-ribosyl groups are transferred to the 2'-position of the terminal adenosine moiety, building up a polymer with an average chain length of 20-30 units.
**PDCD5**	-2,13695	0,001672	Programmed cell death protein 5; May function in the process of apoptosis.
**PHB2**	-3,09409	7,37E-10	Prohibitin-2; Acts as a mediator of transcriptional repression by nuclear hormone receptors via recruitment of histone deacetylases. Functions as an estrogen receptor (ER)-selective coregulator that potentiates the inhibitory activities of antiestrogens and represses the activity of estrogens. Competes with NCOA1 for modulation of ER

			transcriptional activity. In mitochondria, regulates cytochrome-c oxidase assembly (COX) and mitochondrial respiration. Binding to sphingoid 1-phosphate (SPP) modulates its regulator activity.
**PLD6**	-2,24505	0,024744	Mitochondrial cardiolipin hydrolase; Endonuclease that plays a critical role in PIWI-interacting RNA (piRNA) biogenesis during spermatogenesis. piRNAs provide essential protection against the activity of mobile genetic elements.
**PNPT1**	-2,49377	1,92E-06	Polyribonucleotide nucleotidyltransferase 1, mitochondrial; RNA-binding protein implicated in numerous RNA metabolic processes. Catalyzes the phosphorolysis of single-stranded polyribonucleotides processively in the 3'-to-5' direction. Mitochondrial intermembrane factor with RNA-processing exoribonulease activity. Component of the mitochondrial degradosome (mtEXO) complex, that degrades 3' overhang double-stranded RNA with a 3'-to-5' directionality in an ATP-dependent manner. Involved in the degradation of non-coding mitochondrial transcripts (MT-ncRNA) and tRNA-like molecules.
**PPARGC1 A**	-2,85388	3,47E-08	Peroxisome proliferator-activated receptor gamma coactivator 1-alpha; Transcriptional coactivator for steroid receptors and nuclear receptors. Greatly increases the transcriptional activity of PPARG and thyroid hormone receptor on the uncoupling protein promoter. Can regulate key mitochondrial genes that contribute to the program of adaptive thermogenesis. Plays an essential role in metabolic reprogramming in response to dietary availability through coordination of the expression of a wide array of genes involved in glucose and fatty acid metabolism.
**PRIMPOL**	-2,47394	0,000695	DNA-directed primase/polymerase protein; DNA primase and DNA polymerase required to tolerate replication-stalling lesions by bypassing them. Required to facilitate mitochondrial and nuclear replication fork progression by initiating de novo DNA synthesis using dNTPs and acting as an error-prone DNA polymerase able to bypass certain DNA lesions. Shows a high capacity to tolerate DNA damage lesions such as 8oxoG and abasic sites in DNA.
**PTCD2**	-4,63616	1,6E-07	Pentatricopeptide repeat-containing protein 2, mitochondrial; Involved in mitochondrial RNA maturation and mitochondrial respiratory chain function.
**RAB29**	-2,4972	1,65E-06	Ras-related protein Rab-7L1; The small GTPases Rab are key regulators in vesicle trafficking. Essential for maintaining the integrity of the endosome-trans-Golgi network structure. Together with LRRK2, plays a role in the retrograde trafficking pathway for recycling proteins, such as mannose 6 phosphate receptor (M6PR), between lysosomes and the Golgi apparatus in a retromer-dependent manner.
**SAMM50**	-2,13496	2,65E-05	Sorting and assembly machinery component 50 homolog; Plays a crucial role in the maintenance of the structure of mitochondrial cristae and the proper assembly of the mitochondrial respiratory chain complexes. Required for the assembly of TOMM40 into the TOM complex.
**SARS2**	-2,40697	1,46E-05	Serine-tRNA ligase, mitochondrial; Catalyzes the attachment of serine to tRNA(Ser). Is also probably able to aminoacylate tRNA(Sec) with serine, to form the misacylated tRNA L-seryl-tRNA(Sec), which will be further converted into selenocysteinyl-tRNA(Sec).
**SCO1**	-2,07024	0,000193	SCO1 homolog, mitochondrial; Copper metallochaperone essential for the maturation of cytochrome c oxidase subunit II (MT-CO2/COX2). Not required for the synthesis of MT-CO2/COX2 but plays a crucial role in stabilizing MT- CO2/COX2 during its subsequent maturation. Involved in transporting copper to the Cu(A) site on MT-CO2/COX2. Plays an important role in the regulation of copper homeostasis by controlling the abundance and cell membrane localization of copper transporter CTR1.
**SDHAF4**	-4,56394	9,63E-05	Succinate dehydrogenase assembly factor 4, mitochondrial; Plays an essential role in the assembly of succinate dehydrogenase (SDH), an enzyme complex (also referred to as respiratory complex II) that is a component of both the tricarboxylic acid (TCA) cycle and the mitochondrial electron transport chain, and which couples the oxidation of succinate to fumarate with the reduction of ubiquinone (coenzyme Q) to ubiquinol. Binds to the flavoprotein subunit SDHA in its FAD-bound form, blocking the generation of excess reactive oxigen species (ROS).
**SDHD**	-2,03662	0,000286	Succinate dehydrogenase [ubiquinone] cytochrome b small subunit, mitochondrial; Membrane-anchoring subunit of succinate dehydrogenase (SDH) that is involved in complex II of the mitochondrial electron transport chain and is responsible for transferring electrons from succinate to ubiquinone (coenzyme Q).
**SLC25A12**	-4,65039	5,06E-14	Calcium-binding mitochondrial carrier protein Aralar1; Mitochondrial and calcium-binding carrier that catalyzes the calcium-dependent exchange of cytoplasmic glutamate with mitochondrial aspartate across the mitochondrial inner membrane. May have a function in the urea cycle.
**SLC25A13**	-4,66612	1,08E-08	Calcium-binding mitochondrial carrier protein Aralar2; Mitochondrial and calcium-binding carrier that catalyzes the calcium-dependent exchange of cytoplasmic glutamate with mitochondrial aspartate across the mitochondrial inner membrane. May have a function in the urea cycle.
**SLIRP**	-2,99629	3,37E-08	SRA stem-loop-interacting RNA-binding protein, mitochondrial; RNA-binding protein that acts as a nuclear receptor corepressor. Probably acts by binding the SRA RNA, and repressing the SRA-mediated nuclear receptor coactivation. Binds the STR7 loop of SRA RNA. Also able to repress glucocorticoid (GR), androgen (AR), thyroid (TR) and VDR-mediated transactivation.
**SSBP1**	-2,36588	0,000302	Single-stranded DNA-binding protein, mitochondrial; Binds preferentially and cooperatively to pyrimidine rich single-stranded DNA (ss-DNA). In vitro, required to maintain the copy number of mitochondrial DNA (mtDNA) and plays crucial roles during mtDNA replication that stimulate activity of the replisome components POLG and TWNK at the replication fork. Promotes the activity of the gamma complex polymerase POLG, largely by organizing the template DNA and eliminating secondary structures to favor ss-DNA conformations that facilitate POLG activity.
**STAT2**	-3,55984	4,2E-10	Signal transducer and activator of transcription 2; Signal transducer and activator of transcription that mediates signaling by type I IFNs (IFN-alpha and IFN-beta). Following type I IFN binding to cell surface receptors, Jak kinases (TYK2 and JAK1) are activated, leading to tyrosine phosphorylation of STAT1 and STAT2.
**STOML2**	-2,28322	6,03E-06	Stomatin-like protein 2, mitochondrial; Mitochondrial protein that probably regulates the biogenesis and the activity of mitochondria. Stimulates cardiolipin biosynthesis, binds cardiolipin-enriched membranes where it recruits and stabilizes some proteins including prohibitin and may therefore act in the organization of functional microdomains in mitochondrial membranes. Through regulation of the mitochondrial function may play a role into several biological processes including cell migration, cell proliferation, T-cell activation, calcium homeostasis and cellular response to stress.
**TFB2M**	-2,54801	1,56E-05	Dimethyladenosine transferase 2, mitochondrial; S-adenosyl-L-methionine-dependent rRNA methyltransferase which may methylate two specific adjacent adenosines in the loop of a conserved hairpin near the 3'-end of 12S

			mitochondrial rRNA (Probable). Component of the mitochondrial transcription initiation complex, composed at least of TFB2M, TFAM and POLRMT that is required for basal transcription of mitochondrial DNA.
**TIMM10**	-2,186	0,000485	Mitochondrial import inner membrane translocase subunit Tim10; Mitochondrial intermembrane chaperone that participates in the import and insertion of multi-pass transmembrane proteins into the mitochondrial inner membrane. May also be required for the transfer of beta-barrel precursors from the TOM complex to the sorting and assembly machinery (SAM complex) of the outer membrane. Acts as a chaperone-like protein that protects the hydrophobic precursors from aggregation and guide them through the mitochondrial intermembrane space. Belongs to the small Tim family.
**TIMM17A**	-2,13033	8,64E-05	Mitochondrial import inner membrane translocase subunit Tim17-A; Essential component of the TIM23 complex, a complex that mediates the translocation of transit peptide-containing proteins across the mitochondrial inner membrane.
**TIMM23B**	-2,36418	0,001622	Translocase of inner mitochondrial membrane 23 homolog B.
**TIMM50**	-2,14702	2,33E-05	Mitochondrial import inner membrane translocase subunit TIM50; Essential component of the TIM23 complex, a complex that mediates the translocation of transit peptide-containing proteins across the mitochondrial inner membrane. Has some phosphatase activity in vitro; however such activity may not be relevant in vivo.
**TIMM8A**	-4,85484	3,94E-08	Mitochondrial import inner membrane translocase subunit Tim8 A; Mitochondrial intermembrane chaperone that participates in the import and insertion of some multi-pass transmembrane proteins into the mitochondrial inner membrane. Also required for the transfer of beta-barrel precursors from the TOM complex to the sorting and assembly machinery (SAM complex) of the outer membrane. Acts as a chaperone-like protein that protects the hydrophobic precursors from aggregation and guide them through the mitochondrial intermembrane space.
**TIMM9**	-3,66839	7,94E-06	Mitochondrial import inner membrane translocase subunit Tim9; Mitochondrial intermembrane chaperone that participates in the import and insertion of multi-pass transmembrane proteins into the mitochondrial inner membrane. May also be required for the transfer of beta-barrel precursors from the TOM complex to the sorting and assembly machinery (SAM complex) of the outer membrane. Acts as a chaperone-like protein that protects the hydrophobic precursors from aggregation and guide them through the mitochondrial intermembrane space. Belongs to the small Tim family.
**TMEM126 A**	-4,88827	4,72E-09	Transmembrane protein 126A; Belongs to the TMEM126 family.
**TMEM126 B**	-4,44545	5,65E-11	Complex I assembly factor TMEM126B, mitochondrial; Chaperone protein involved in the assembly of the mitochondrial NADH:ubiquinone oxidoreductase complex (complex I). Participates in constructing the membrane arm of complex I. Belongs to the TMEM126 family.
**TMEM223**	-2,11974	0,000238	Transmembrane protein 223.
**TNFSF10**	-4,68408	3,33E-07	Tumor necrosis factor ligand superfamily member 10; Cytokine that binds to TNFRSF10A/TRAILR1, TNFRSF10B/TRAILR2, TNFRSF10C/TRAILR3, TNFRSF10D/TRAILR4 and possibly also to TNFRSF11B/OPG. Induces apoptosis. Its activity may be modulated by binding to the decoy receptors TNFRSF10C/TRAILR3, TNFRSF10D/TRAILR4 and TNFRSF11B/OPG that cannot induce apoptosis. Belongs to the tumor necrosis factor family.
**TOMM22**	-2,31043	7,68E-06	Mitochondrial import receptor subunit TOM22 homolog; Central receptor component of the translocase of the outer membrane of mitochondria (TOM complex) responsible for the recognition and translocation of cytosolically synthesized mitochondrial preproteins. Together with the peripheral receptor TOM20 functions as the transit peptide receptor and facilitates the movement of preproteins into the translocation pore. Required for the translocation across the mitochondrial outer membrane of cytochrome P450 monooxygenases.
**TOMM40L**	-3,28068	1,4E-07	Mitochondrial import receptor subunit TOM40B; Potential channel-forming protein implicated in import of protein precursors into mitochondria; Belongs to the Tom40 family.
**TOMM6**	-2,86094	1,78E-08	Mitochondrial import receptor subunit TOM6 homolog; Translocase of outer mitochondrial membrane 6.
**TOMM70**	-2,00116	0,000992	Mitochondrial import receptor subunit TOM70; Receptor that accelerates the import of all mitochondrial precursor proteins.
**TRMT5**	-2,87415	2,91E-05	tRNA (guanine(37)-N1)-methyltransferase; Involved in mitochondrial tRNA methylation. Specifically methylates the N1 position of guanosine-37 in various tRNAs. Methylation is not dependent on the nature of the nucleoside 5' of the target nucleoside. This is the first step in the biosynthesis of wybutosine (yW), a modified base adjacent to the anticodon of tRNAs and required for accurate decoding.
**TRMT61B**	-2,06339	0,001422	tRNA (adenine(58)-N(1))-methyltransferase, mitochondrial; Methyltransferase that catalyzes the formation of N(1)- methyladenine at position 58 (m1A58) in various tRNAs in mitochondrion, including tRNA(Leu) (deciphering codons UUA or UUG), tRNA(Lys) and tRNA(Ser) (deciphering codons UCA, UCU, UCG or UCC). Catalyzes the formation of 1-methyladenosine at position 947 of mitochondrial 16S ribosomal RNA and this modification is most likely important for mitoribosomal structure and function.
**TRNT1**	-2,28341	2,87E-05	CCA tRNA nucleotidyltransferase 1, mitochondrial; [Isoform 1]: Adds and repairs the conserved 3'-CCA sequence necessary for the attachment of amino acids to the 3' terminus of tRNA molecules, using CTP and ATP as substrates.
**TYMP**	-2,14186	0,033345	Thymidine phosphorylase; May have a role in maintaining the integrity of the blood vessels. Has growth promoting activity on endothelial cells, angiogenic activity in vivo and chemotactic activity on endothelial cells in vitro.
**UQCC2**	-2,26682	3,5E-05	Ubiquinol-cytochrome-c reductase complex assembly factor 2; Required for the assembly of the ubiquinol-cytochrome c reductase complex (mitochondrial respiratory chain complex III or cytochrome b-c1 complex). Plays a role in the modulation of respiratory chain activities such as oxygen consumption and ATP production.
**UQCR10**	-2,36266	1,83E-05	Cytochrome b-c1 complex subunit 9; Component of the ubiquinol-cytochrome c oxidoreductase, a multisubunit transmembrane complex that is part of the mitochondrial electron transport chain which drives oxidative phosphorylation.
**WARS2**	-2,81538	5,96E-07	Tryptophan--tRNA ligase, mitochondrial; Mitochondrial aminoacyl-tRNA synthetase that activate and transfer the amino acids to their corresponding tRNAs during the translation of mitochondrial genes and protein synthesis.

**YARS2**	-2,227	5,43E-05	Tyrosine-tRNA ligase, mitochondrial; Catalyzes the attachment of tyrosine to tRNA(Tyr) in a two- step reaction: tyrosine is first activated by ATP to form Tyr-AMP and then transferred to the acceptor end of tRNA(Tyr). Belongs to the class-I aminoacyl-tRNA synthetase family.

Supp. Table S5. GO up-regulated genes COMB. vs CTR.

**GO.ID**	**Description**	**FDR**	**Genes**
**GO:0007399**	**nervous system development**	0.000000085	DPYSL4,CLDN3,VLDLR,NEFL,PTPRF,RORA,KDM7A,C3ORF70,NPAS1,FGF11,BHLHE41,BNIP3,KCTD11,BHLHE40,VEGFA,DDIT4,SPINT1,NEDD4L,CPNE5,INA,ADM,RGS2,CLCF1,DKK1,CYFIP2,ABCB6,MFSD2A,LOXL3,NOTCH3,UNC119,TBX1,PKD1,PPP2R5B,UGCG,PHGDH,EFNA3,BAIAP2,TPBG,MAPT,KDM2B,CKB,FZD1,SGK1,CHAC1,SLC2A1,SH2B2,VEGFC,FZD3,APLP1,LRIG1,ACHE,FZD7,BRSK2,SLC7A5,TENM4,JAG2,KLF4,NTM,RBPJ,ETV5,MAP2K1,TENM2,RAP2A,WNT11,SPHK1,NDRG1,SDK1,NEDD4,EGR2,TUBB3,HSPG2,TSKU,MAP6,NPTX1,ARC,NPHP4,LIF,APBA2,TRIO,IGF1R,SLC1A1,ABL1,IER2,SPRY3,MEF2A,RAPGEFL1,ARHGAP35,ECE1,KDM4B,EPHB1,CELSR1,WEE1,NCKIPSD,ENC1,NAB2,LRP12,IRX5,DNER,STXBP1,CSPG4,SEMA7A,BOC,ND4,PLXNB3,DPCD,AATK,HLX,AGRN,RTN4,ACAP3,ICAM5,WNT4,PCSK9,NCOR2,DEGS1,PLXNA3,,DPYSL2,NGF,EDN1,,ADARB1,OBSCN,,SPTBN4,NCAM1,RUNX1,CHRM2,S100A10,CEP85L,TRPV4,MDGA1,IRS2,HES6,PLK5,NAV1,CLSTN1,ABCA2,SH3RF1,SUN1,PPARD,MET,SEMA4F,NR2F1,NR4A2,MYLIP,SLC5A3,,NGEF,MAFB,NRXN3,BOK,GPR157,SEMA4B,TUBB2B,,IGSF8,DCLK2,PHF10,EFNB2,LDLR,CRABP2,NTRK3,PLEC,RAP1GAP,EML1,AGTPBP1,ZMIZ1,SPOCK1,GPC1,WNT5B,ADGRG1,ARF6,MEIS3,NGFR,CASP3,CIC,MACO1,UNC5B,NRTN,,TLR4,PDLIM5,SDK2,RAB6B,LRP1,ARHGEF10,COL4A1,KIRREL3,TSC22D4,HSPA5,MICALL1,OGT,APAF1,PTEN,CDK6,PTPRM,GPRIN1,WNT2,,PSD,EPB41L3,NANOS1,TWF1,JAK2,ANXA2,NTN4,FOXD1,EGFR,STK25,FLNA,,RTN4R,KCNIP2,,HES4,BRD2,ABL2,SYNDIG1,NEGR1,SEMA6D,DGKG,N4BP3,SCARB2,ARHGAP32,BCR,TRNP1,SOX8,KDM6B,NPC1,BCL10,WDR47,SH3GL1,GSK3B,,AXL,TNXB,GIT1,VTN,RERE,ATXN1,NR2F6,SKIL,AKT3,SEMA4G,HS6ST1,CD320,ITGB4,ATP1B2,TIAM1,SIX4,STK24,ZSWIM6,TNIK,CRIM1,PPP2R3A,GDPD5,ARHGEF2,INKA1,TANC2,KLF7,CDH2,NTN1,BRCA2,EPHB2,TBX3,EPHA2,CASZ1,PRDM6,MMP24,COLQ,SPHK2,E2F1,IL1RAP,SERPINI1,JARID2,CSNK2A2,NAV2,PLAG1,RAPGEF2,CELSR2,EPOP,MAPK8IP2,IL15RA,SS18L1,FZD8,TAL2,RGS4,RAB3A,EOMES,NPHP3,PPP3R1,SHROOM2,SOX9,PCDH1,SOX13,NRCAM,LARGE1,ITGA6,DTNB,SHROOM4,STMN3,ACVR1B,SYN1,ALDH1A3,TSPAN2,NEURL1,SEMA3F,AMIGO3,CLSTN2,PDZD7,CYP46A1,FZD5,IRX3,ADCY1,PLXNA4,HEY1,SLC1A2,SYNGR3,LRRC4B,MDGA2,MYRF,SATB2,B3GNT5,TMOD2,PTPRO,GPRC5B,FSCN2,WNT9A,TP73,RND1,UNC13A,VAX2,LAMA1,TTLL7
**GO:0022008**	**neurogenesis**	0.000146	VLDLR,NEFL,PTPRF,RORA,BHLHE41,BNIP3,KCTD11,BHLHE40,VEGFA,DDIT4,SPINT1,NEDD4L,CPNE5,ADM,RGS2,CLCF1,DKK1,CYFIP2,MFSD2A,NOTCH3,PPP2R5B,UGCG,PHGDH,EFNA3,BAIAP2,TPBG,MAPT,FZD1,SGK1,CHAC1,VEGFC,FZD3,APLP1,FZD7,,BRSK2,SLC7A5,TENM4,JAG2,KLF4,NTM,RBPJ,ETV5,MAP2K1,TENM2,RAP2A,WNT11,NDRG1,SDK1,NEDD4,EGR2,TUBB3,TSKU,MAP6,NPTX1,ARC,NPHP4,LIF,TRIO,IGF1R,SLC1A1,ABL1,IER2,SPRY3,MEF2A,ARHGAP35,ECE1,EPHB1,CELSR1,WEE1,NCKIPSD,ENC1,NAB2,LRP12,IRX5,DNER,STXBP1,CSPG4,SEMA7A,BOC,PLXNB3,AGRN,RTN4,ACAP3,WNT4,PCSK9,PLXNA3,,DPYSL2,NGF,EDN1,ADARB1,OBSCN,,SPTBN4,NCAM1,RUNX1,S100A10,CEP85L,TRPV4,MDGA1,HES6,PLK5,NAV1,ABCA2,SH3RF1,SUN1,MET,SEMA4F,NR2F1,NR4A2,MYLIP,NGEF,NRXN3,BOK,GPR157,SEMA4B,TUBB2B,,DCLK2,EFNB2,LDLR,CRABP2,NTRK3,PLEC,RAP1GAP,EML1,AGTPBP1,ZMIZ1,SPOCK1,GPC1,WNT5B,ADGRG1,ARF6,NGFR,CASP3,UNC5B,NRTN,,TLR4,PDLIM5,SDK2,RAB6B,LRP1,ARHGEF10,KIRREL3,TSC22D4,MICALL1,PTEN,CDK6,PTPRM,GPRIN1,WNT2,PSD,EPB41L3,NANOS1,TWF1,JAK2,ANXA2,NTN4,FOXD1,EGFR,STK25,FLNA,,RTN4R,KCNIP2,,ABL2,NEGR1,SEMA6D,DGKG,SCARB2,ARHGAP32,SOX8,NPC1,WDR47,GSK3B,,AXL,TNXB,GIT1,VTN,RERE,NR2F6,SKIL,SEMA4G,HS6ST1,ITGB4,ATP1B2,TIAM1,SIX4,STK24,ZSWIM6,TNIK,PPP2R3A,GDPD5,ARHGEF2,TANC2,KLF7,CDH2,NTN1,EPHB2,EPHA2,CASZ1,PRDM6,MMP24,SERPINI1,NAV2,PLAG1,RAPGEF2,CELSR2,EPOP,MAPK8IP2,IL15RA,SS18L1,FZD8,RAB3A,EOMES,PPP3R1,SOX9,SOX13,NRCAM,LARGE1,ITGA6,DTNB,STMN3,SYN1,TSPAN2,NEURL1,SEMA3F,AMIGO3,PDZD7,FZD5,IRX3,ADCY1,PLXNA4,HEY1,MDGA2,MYRF,SATB2,PTPRO,GPRC5B,FSCN2,WNT9A,TP73,RND1,UNC13A,VAX2,LAMA1
**GO:0050808**	**synapse organization**	0.0000016569160201305067	SNCB,NEFL,PTPRF,PPFIA4,EGLN1,HOMER1,INA,DKK1,BAIAP2,TPBG,MAPT,SNTA1,ARHGAP22,FZD1,ACHE,RAP2A,SDK1,NEDD4,MPP2,PPFIA3,NPTX1,INSR,ARC,IGF1R,SLC1A1,ABL1,F2R,CTTNBP2,EPHB1,SYNPO,DNER,ACTN1,AGRN,ICAM5,SPTBN4,SLC18A3,SPTB,FRRS1L,MDGA1,CLSTN1,NGEF,NRXN3,EFNB2,ARF6,PDLIM5,SDK2,COL4A1,KIRREL3,OGT,PTEN,FLNA,ITGB3,SYNDIG1,NEGR1,PDZRN3,NEDD9,TIAM1,SIX4,TANC2,CDH2,NTN1,EPHB2,COLQ,IL1RAP,RAB3A,RHOB,NRCAM,LARGE1,SYN1,NEURL1,SEMA3F,AMIGO3,CLSTN2,FZD5,PLXNA4,LRRC4B,PTPRO,UNC13A
**GO:0099536**	**synaptic signaling**	0.0000017640861493478906	SNCB,NEFL,NR4A1,AKAP12,SLC6A6,PRR7,GABRE,HOMER1,INA,DKK1,CHRM4,HRH1,UNC119,BAIAP2,TPBG,MAPT,HTR7,ADCY8,GRIN3B,ACHE,PLAT,DTNA,EGR2,MPP2,PTGS2,PPFIA3,NPTX1,ARC,APBA2,TRIO,SLC1A1,ABL1,F2R,PIP5K1C,EPHB1,ADORA2B,STXBP1,ADRB2,SLC6A9,AGRN,RTN4,DNAJC5,STX1A,KCMF1,EDN1,ADARB1,RAB3B,SLC18A3,CHRM2,GLS,RNF19A,FRRS1L,CLSTN1,PLK2,GALR3,NRXN3,TUBB2B,GRIA3,BAIAP3,STX11,VDAC1,DLGAP4,NGFR,DYSF,PTEN,ITPR1,NPTX2,JAK2,GPR176,SYT11,KCNIP2,CHRNE,KCNMB4,SLC1A4,BCR,RASD2,GSK3B,GIT1,BEGAIN,CDH2,EPHB2,SORCS2,COLQ,LIN7B,IL1RAP,RAPGEF2,MAPK8IP2,NPTXR,RGS4,RAB3A,PPP3R1,MIR324,LARGE1,INSYN1,DTNB,PLCB4,GJC1,SYN1,NEURL1,CRHR2,GABRR2,CLSTN2,CYP46A1,KCNC4,ADCY1,HCRTR1,SLC1A2,CACNG8,TMOD2,UNC13A
**GO:0048667**	**cell morphogenesis involved in neuron differentiation**	0.000002130565684892942	VLDLR,NEFL,VEGFA,NEDD4L,CYFIP2,MFSD2A,NOTCH3,EFNA3,BAIAP2,TPBG,MAPT,FZD3,APLP1,BRSK2,MAP2K1,RAP2A,NEDD4,EGR2,TUBB3,TSKU,MAP6,NPTX1,ARC,TRIO,IGF1R,ABL1,MEF2A,ARHGAP35,ECE1,EPHB1,STXBP1,SEMA7A,BOC,PLXNB3,RTN4,PLXNA3,,NGF,EDN1,ADARB1,OBSCN,,SPTBN4,NCAM1,SEMA4F,NR4A2,NGEF,NRXN3,SEMA4B,TUBB2B,EFNB2,CRABP2,NGFR,UNC5B,,PDLIM5,PTEN,PTPRM,FOXD1,STK25,RT

			N4R,SEMA6D,ARHGAP32,WDR47,GSK3B,,RERE,SKIL,SEMA4G,TIAM1,TNIK,TANC2,KLF7,CDH2,NTN1,EPHB2,RAPGEF2,CELSR2,MAPK8IP2,SS18L1,RAB3A,NRCAM,SEMA3F,PDZD7,ADCY1,PLXNA4,PTPRO,VAX2
**GO:0048812**	**neuron projection morphogenesis**	0.0000035636513981676303	VLDLR,NEFL,VEGFA,NEDD4L,CPNE5,CYFIP2,MFSD2A,NOTCH3,EFNA3,BAIAP2,TPBG,MAPT,SGK1,FZD3,APLP1,BRSK2,MAP2K1,RAP2A,NEDD4,EGR2,TUBB3,TSKU,MAP6,NPTX1,ARC,TRIO,IGF1R,ABL1,SPRY3,MEF2A,ARHGAP35,ECE1,EPHB1,WEE1,STXBP1,SEMA7A,BOC,PLXNB3,RTN4,PLXNA3,,NGF,EDN1,ADARB1,OBSCN,,SPTBN4,NCAM1,SEMA4F,NR4A2,NGEF,NRXN3,SEMA4B,TUBB2B,EFNB2,CRABP2,NGFR,UNC5B,,PDLIM5,KIRREL3,TSC22D4,PTEN,PTPRM,EPB41L3,FOXD1,STK25,RTN4R,SEMA6D,ARHGAP32,WDR47,GSK3B,RERE,SKIL,SEMA4G,TIAM1,TNIK,TANC2,KLF7,CDH2,NTN1,EPHB2,RAPGEF2,CELSR2,MAPK8IP2,SS18L1,RAB3A,NRCAM,SEMA3F,ADCY1,PLXNA4,PTPRO,UNC13A,VAX2
**GO:0099537**	**trans-synaptic signaling**	0.000009373408512360632	SNCB,NEFL,NR4A1,AKAP12,SLC6A6,GABRE,HOMER1,INA,DKK1,CHRM4,HRH1,UNC119,BAIAP2,TPBG,MAPT,HTR7,ADCY8,GRIN3B,ACHE,PLAT,DTNA,EGR2,MPP2,PTGS2,PPFIA3,NPTX1,ARC,APBA2,TRIO,SLC1A1,ABL1,F2R,PIP5K1C,EPHB1,ADORA2B,STXBP1,ADRB2,SLC6A9,RTN4,DNAJC5,STX1A,EDN1,ADARB1,RAB3B,SLC18A3,CHRM2,GLS,RNF19A,FRRS1L,CLSTN1,PLK2,GALR3,NRXN3,TUBB2B,GRIA3,BAIAP3,STX11,VDAC1,DLGAP4,NGFR,DYSF,PTEN,NPTX2,JAK2,GPR176,SYT11,KCNIP2,CHRNE,KCNMB4,SLC1A4,BCR,RASD2,GSK3B,GIT1,BEGAIN,CDH2,EPHB2,SORCS2,COLQ,LIN7B,IL1RAP,RAPGEF2,MAPK8IP2,NPTXR,RGS4,RAB3A,PPP3R1,MIR324,LARGE1,INSYN1,PLCB4,GJC1,SYN1,NEURL1,CRHR2,GABRR2,CLSTN2,CYP46A1,KCNC4,ADCY1,HCRTR1,SLC1A2,CACNG8,TMOD2,UNC13A
**GO:0007420**	**brain development**	0.000009755716343133854	NEFL,RORA,KDM7A,BNIP3,DDIT4,INA,DKK1,ABCB6,MFSD2A,NOTCH3,PHGDH,KDM2B,CKB,FZD1,SLC2A1,FZD3,APLP1,RBPJ,MAP2K1,SPHK1,EGR2,HSPG2,TSKU,IGF1R,SLC1A1,ABL1,ARHGAP35,KDM4B,EPHB1,SEMA7A,ND4,DPCD,AATK,RTN4,WNT4,NCOR2,PLXNA3,MDGA1,IRS2,SUN1,NR4A2,,MAFB,TUBB2B,DCLK2,EML1,AGTPBP1,ZMIZ1,ADGRG1,MEIS3,CASP3,CIC,MACO1,COL4A1,KIRREL3,HSPA5,APAF1,PTEN,CDK6,WNT2,,NANOS1,EGFR,FLNA,,RTN4R,,SEMA6D,ARHGAP32,BCR,TRNP1,KDM6B,WDR47,GSK3B,,AXL,GIT1,RERE,ATXN1,AKT3,ATP1B2,ZSWIM6,CDH2,BRCA2,EPHB2,TBX3,SPHK2,E2F1,CSNK2A2,RAPGEF2,CELSR2,TAL2,EOMES,SHROOM2,NRCAM,LARGE1,SHROOM4,ALDH1A3,AMIGO3,PLXNA4,SLC1A2,SYNGR3,B3GNT5,VAX2
**GO:0098916**	**anterograde trans-synaptic signaling**	0.000012135686064176593	SNCB,NEFL,NR4A1,AKAP12,SLC6A6,GABRE,HOMER1,INA,DKK1,CHRM4,HRH1,UNC119,BAIAP2,TPBG,MAPT,HTR7,ADCY8,GRIN3B,ACHE,PLAT,DTNA,EGR2,MPP2,PTGS2,PPFIA3,NPTX1,ARC,APBA2,TRIO,SLC1A1,ABL1,F2R,PIP5K1C,EPHB1,ADORA2B,STXBP1,ADRB2,SLC6A9,RTN4,DNAJC5,STX1A,EDN1,ADARB1,RAB3B,SLC18A3,CHRM2,GLS,RNF19A,FRRS1L,CLSTN1,PLK2,GALR3,NRXN3,TUBB2B,GRIA3,BAIAP3,STX11,VDAC1,DLGAP4,NGFR,DYSF,PTEN,NPTX2,JAK2,GPR176,SYT11,KCNIP2,CHRNE,KCNMB4,SLC1A4,BCR,RASD2,GSK3B,GIT1,BEGAIN,CDH2,EPHB2,SORCS2,COLQ,LIN7B,RAPGEF2,MAPK8IP2,NPTXR,RGS4,RAB3A,PPP3R1,MIR324,LARGE1,INSYN1,PLCB4,GJC1,SYN1,NEURL1,CRHR2,GABRR2,CLSTN2,CYP46A1,KCNC4,ADCY1,HCRTR1,SLC1A2,CACNG8,TMOD2,UNC13A
**GO:0007268**	**chemical synaptic transmission**	0.000012135686064176593	SNCB,NEFL,NR4A1,AKAP12,SLC6A6,GABRE,HOMER1,INA,DKK1,CHRM4,HRH1,UNC119,BAIAP2,TPBG,MAPT,HTR7,ADCY8,GRIN3B,ACHE,PLAT,DTNA,EGR2,MPP2,PTGS2,PPFIA3,NPTX1,ARC,APBA2,TRIO,SLC1A1,ABL1,F2R,PIP5K1C,EPHB1,ADORA2B,STXBP1,ADRB2,SLC6A9,RTN4,DNAJC5,STX1A,EDN1,ADARB1,RAB3B,SLC18A3,CHRM2,GLS,RNF19A,FRRS1L,CLSTN1,PLK2,GALR3,NRXN3,TUBB2B,GRIA3,BAIAP3,STX11,VDAC1,DLGAP4,NGFR,DYSF,PTEN,NPTX2,JAK2,GPR176,SYT11,KCNIP2,CHRNE,KCNMB4,SLC1A4,BCR,RASD2,GSK3B,GIT1,BEGAIN,CDH2,EPHB2,SORCS2,COLQ,LIN7B,RAPGEF2,MAPK8IP2,NPTXR,RGS4,RAB3A,PPP3R1,MIR324,LARGE1,INSYN1,PLCB4,GJC1,SYN1,NEURL1,CRHR2,GABRR2,CLSTN2,CYP46A1,KCNC4,ADCY1,HCRTR1,SLC1A2,CACNG8,TMOD2,UNC13A
**GO:0002521**	**leukocyte differentiation**	0.00007181568288905587	RORA,OSCAR,KLF10,GPR137B,UBASH3B,MT1G,HLA-G,PRR7,VEGFA,FSTL3,CLCF1,PRTN3,LOXL3,TFRC,FZD7,JAG2,RBPJ,SHB,HLA-B,CEBPG,JUNB,ZBTB1,NFIL3,SH3PXD2A,MFNG,CCN4,JMJD6,LIF,ABL1,GPR68,TPD52,RASSF2,IRF7,PCK1,INHA,NFKBIZ,HLX,WNT4,RUNX1,F2RL1,TNFSF13B,LRRC17,EGR1,FOXO3,TNFSF9,SH3RF1,ARID2,FAM20C,MAFB,CEACAM1,NFKBID,PHF10,ZMIZ1,TET2,TLR4,TF,CDK6,ANXA2,,,ARMC5,SOCS1,NEDD9,AXL,TRIB1,FASN,CAMK4,EPHA2,IRF4,IL4R,IL15RA,FZD8,EOMES,GAB3,INPP5D,SOX13,SBNO2,LARGE1,GAB2,LFNG,TSPAN2,IFNE,FZD5,KITLG,CARD11,RELB,RAG1
**GO:0061564**	**axon development**	0.00008223555075630958	NEFL,PTPRF,VEGFA,CYFIP2,NOTCH3,EFNA3,BAIAP2,MAPT,FZD3,APLP1,BRSK2,KLF4,MAP2K1,EGR2,TUBB3,TSKU,MAP6,NPTX1,TRIO,IGF1R,ABL1,ARHGAP35,ECE1,EPHB1,STXBP1,SEMA7A,BOC,PLXNB3,RTN4,PLXNA3,,NGF,EDN1,ADARB1,OBSCN,,SPTBN4,NCAM1,SEMA4F,NR4A2,NRXN3,SEMA4B,TUBB2B,EFNB2,CRABP2,NGFR,CASP3,UNC5B,,PTEN,PTPRM,JAK2,FOXD1,STK25,FLNA,RTN4R,SEMA6D,ARHGAP32,WDR47,GSK3B,SKIL,SEMA4G,TIAM1,STK24,KLF7,CDH2,NTN1,EPHB2,RAB3A,NRCAM,TSPAN2,SEMA3F,ADCY1,PLXNA4,PTPRO,VAX2
**GO:0050804**	**modulation of chemical synaptic transmission**	0.0001088386744842272	NEFL,AKAP12,SLC6A6,HOMER1,INA,DKK1,HRH1,BAIAP2,MAPT,ADCY8,GRIN3B,ACHE,PLAT,EGR2,MPP2,PTGS2,PPFIA3,NPTX1,ARC,APBA2,TRIO,SLC1A1,ABL1,F2R,EPHB1,ADORA2B,STXBP1,ADRB2,SLC6A9,RTN4,STX1A,EDN1,SLC18A3,CHRM2,RNF19A,FRRS1L,CLSTN1,PLK2,NRXN3,TUBB2B,GRIA3,BAIAP3,DLGAP4,NGFR,DYSF,PTEN,NPTX2,JAK2,SYT11,KCNMB4,BCR,GSK3B,GIT1,BEGAIN,CDH2,EPHB2,SORCS2,RAPGEF2,MAPK8IP2,NPTXR,RGS4,RAB3A,PPP3R1,MIR324,LARGE1,PLCB4,SYN1,NEURL1,CRHR2,CLSTN2,CYP46A1,ADCY1,CACNG8,UNC13A
**GO:0007409**	**axonogenesis**	0.00011298725743874879	NEFL,VEGFA,CYFIP2,NOTCH3,EFNA3,BAIAP2,MAPT,FZD3,APLP1,BRSK2,MAP2K1,EGR2,TUBB3,TSKU,MAP6,NPTX1,TRIO,IGF1R,ABL1,ARHGAP35,ECE1,EPHB1,STXBP1,SEMA7A,BOC,PLXNB3,RTN4,PLXNA3,,NGF,EDN1,ADARB1,OBSCN,,SPTBN4,NCAM1,SEMA4F,NR4A2,NRXN3,SEMA4B,TUBB2B,EFNB2,CRABP2,NGFR,UNC5B,,PTEN,PTPRM,FOXD1,STK25,RTN4R,SEMA6D,ARHGAP32,WDR47,GSK3B,SKIL,SEMA4G,TIAM1,KLF7,CDH2,NTN1,EPHB2,RAB3A,NRCAM,SEMA3F,ADCY1,PLXNA4,PTPRO,VAX2

**GO:0099177**	**regulation of trans-synaptic signaling**	0.00011842681419877416	NEFL,AKAP12,SLC6A6,HOMER1,INA,DKK1,HRH1,BAIAP2,MAPT,ADCY8,GRIN3B,ACHE,PLAT,EGR2,MPP2,PTGS2,PPFIA3,NPTX1,ARC,APBA2,TRIO,SLC1A1,ABL1,F2R,EPHB1,ADORA2B,STXBP1,ADRB2,SLC6A9,RTN4,STX1A,EDN1,SLC18A3,CHRM2,RNF19A,FRRS1L,CLSTN1,PLK2,NRXN3,TUBB2B,GRIA3,BAIAP3,DLGAP4,NGFR,DYSF,PTEN,NPTX2,JAK2,SYT11,KCNMB4,BCR,GSK3B,GIT1,BEGAIN,CDH2,EPHB2,SORCS2,RAPGEF2,MAPK8IP2,NPTXR,RGS4,RAB3A,PPP3R1,MIR324,LARGE1,PLCB4,SYN1,NEURL1,CRHR2,CLSTN2,CYP46A1,ADCY1,CACNG8,UNC13A
**GO:0045321**	**leukocyte activation**	0.00021780337397804374	RORA,GPR137B,MT1G,HLA-G,PRR7,RASAL3,CLCF1,LOXL3,MAPT,CDKN1A,TFRC,PAG1,HLA-A,FZD7,JAG2,RBPJ,SHB, CEBPG,JUNB,SPHK1,NDRG1,ZBTB1,NFIL3,MFNG,JMJD6,CXCL8,ABL1,TPD52,SELPLG,EPHB1,ADORA2B,PCK1,STXBP1,INHA,NFKBIZ,HLX,ULBP2,NR4A3,WNT4,HLA-F,EDN1,MFHAS1,HLA-E,RUNX1,SLC7A1,F2RL1,IL12A,TNFSF13B,CAV1,DUSP3,IRS2,EGR1,HLA-DPB1,FOXO3,TNFSF9,SH3RF1,ARID2,MAFB,CEACAM1,NFKBID,PHF10,EFNB2,LDLR,ZMIZ1,HLA-DPA1,CASP3,TLR4,DYSF,CD274,CDK6,TNFAIP3,JAK2,SYT11,,ABL2,WNK4,TNFRSF13C,ULBP1,BCR,ARMC5,SOCS1,BCL10,THBS1,NEDD9,,AXL,DPP4,KMT2E,CAMK4,CD320,EPHB2,TSLP,IRF4,THEMIS2,SPHK2,IL4R,IL15RA,CBLB,FZD8,EOMES,INPP5D,MICB,SOX13,SBNO2,GAB2,LFNG,IL20RB,IFNE,FZD5,SLC11A1,KITLG,CARD11,,UNC13D,RELB,RAG1,PDCD1LG2,
**GO:0002486**	**antigen processing and presentation of endogenous peptide antigen via MHC class I via ER pathway, TAP-independent**	0.0003713873678796384	HLA-G,HLA-A,HLA-B,HLA-C,ULBP2,HLA-F,HLA-E,ULBP1,MICB
**GO:0050807**	**regulation of synapse organization**	0.0007628410699835833	NEFL,EGLN1,HOMER1,DKK1,BAIAP2,TPBG,ARHGAP22,FZD1,RAP2A,NEDD4,ARC,ABL1,CTTNBP2,EPHB1,AGRN,ICAM5,SLC18A3,MDGA1,CLSTN1,NGEF,ARF6,PDLIM5,OGT,PTEN,SYNDIG1,NEGR1,NEDD9,TIAM1,SIX4,TANC2,CDH2,NTN1,EPHB2,COLQ,IL1RAP,RHOB,NRCAM,NEURL1,SEMA3F,AMIGO3,CLSTN2,LRRC4B,PTPRO
**GO:0030099**	**myeloid cell differentiation**	0.000793421076251029	LOX,OSCAR,KLF10,GPR137B,UBASH3B,MT1G,VEGFA,FSTL3,PRTN3,TFRC,RBPJ,,CEBPG,JUNB,SH3PXD2A,CCN4,JMJD6,LIF,RCOR1,GPR68,RASSF2,IRF7,INHA,ACTN1,MFHAS1,RUNX1,SMAP1,F2RL1,LRRC17,ETS1,FOXO3,FAM20C,MAFB,CEACAM1,ZNF385A,CASP3,TET2,TLR4,TF,CDK6,JAK2,ANXA2,FLNA,PTBP3,,RBM15,,SOCS1,NEDD9,TRIB1,FASN,KMT2E,CAMK4,EPHA2,IRF4,GAB3,INPP5D,SBNO2,LARGE1,GAB2,ACVR1B,TSPAN2,KITLG,RELB
**GO:0002428**	**antigen processing and presentation of peptide antigen via MHC class Ib**	0.001836691878143224	HLA-G,HLA-A,HLA-B,HLA-C,ULBP2,HLA-F,HLA-E,ULBP1,MICB
**GO:0007159**	**leukocyte cell-cell adhesion**	0.004914312244945061	HLA-G,CCL28,RASAL3,LOXL3,TFRC,PAG1,HLA-A,KLF4,SHB,ZBTB1,NT5E,ABL1,SELPLG,ITGA5,PCK1,NFKBIZ,HLX,NR4A3,HLA-E,RUNX1,SLC7A1,IL12A,TNFSF13B,CAV1,DUSP3,ETS1,HLA-DPB1,FOXO3,TNFSF9,ARID2,CEACAM1,NFKBID,ELANE,PHF10,EFNB2,ZMIZ1,HLA-DPA1,CASP3,CD274,IRAK1,JAK2,TNIP1,FUT4,,ABL2,TNFRSF13C,SOCS1,BCL10,DPP4,IL4R,CBLB,SOX13,SLC39A8,IL20RB,KITLG,CARD11,RAG1,PDCD1LG2
**GO:0007416**	**synapse assembly**	0.005633047963357944	DKK1,TPBG,MAPT,FZD1,ACHE,RAP2A,SDK1,NPTX1,EPHB1,DNER,AGRN,ICAM5,MDGA1,CLSTN1,NRXN3,EFNB2,ARF6,PDLIM5,SDK2,KIRREL3,OGT,PTEN,SYNDIG1,NEGR1,SIX4,CDH2,NTN1,EPHB2,COLQ,IL1RAP,NRCAM,LARGE1,AMIGO3,CLSTN2,FZD5,LRRC4B
**GO:0051960**	**regulation of nervous system development**	0.006140498431399724	NEFL,BHLHE41,BHLHE40,VEGFA,SPINT1,CLCF1,DKK1,BAIAP2,TPBG,MAPT,VEGFC,FZD3,SLC7A5,TENM4,ETV5,MAP2K1,,EGR2,MAP6,LIF,EPHB1,SEMA7A,PLXNB3,AGRN,RTN4,PLXNA3,NGF,TG,,S100A10,HES6,CLSTN1,SEMA4F,SEMA4B,LDLR,CRABP2,PTEN,WNT2,ANXA2,STK25,RTN4R,,SYNDIG1,SEMA6D,ARHGAP32,SOX8,SKIL,SEMA4G,TIAM1,NTN1,EPHB2,IL1RAP,PLAG1,RAPGEF2,SS18L1,NPHP3,NEURL1,SEMA3F,AMIGO3,CLSTN2,PLXNA4,HEY1,LRRC4B,MYRF,TP73
**GO:1903037**	**regulation of leukocyte cell-cell adhesion**	0.009541293877966784	HLA-G,CCL28,RASAL3,LOXL3,TFRC,PAG1,HLA-A,KLF4,SHB,,ZBTB1,ABL1,PCK1,NFKBIZ,HLX,NR4A3,HLA-E,RUNX1,SLC7A1,IL12A,TNFSF13B,CAV1,DUSP3,ETS1,HLA-DPB1,FOXO3,TNFSF9,ARID2,CEACAM1,NFKBID,ELANE,PHF10,EFNB2,ZMIZ1,HLA-DPA1,CASP3,CD274,IRAK1,JAK2,FUT4,,ABL2,TNFRSF13C,SOCS1,BCL10,DPP4,IL4R,CBLB,SOX13,IL20RB,KITLG,CARD11,RAG1,PDCD1LG2
**GO:0050770**	**regulation of axonogenesis**	0.011465577562018842	NEFL,VEGFA,MAPT,BRSK2,MAP2K1,MAP6,ARHGAP35,SEMA7A,PLXNB3,RTN4,PLXNA3,NGF,,SEMA4F,SEMA4B,CRABP2,PTEN,STK25,RTN4R,SEMA6D,ARHGAP32,GSK3B,SKIL,SEMA4G,TIAM1,CDH2,NTN1,EPHB2,SEMA3F,PLXNA4
**GO:0051251**	**positive regulation of lymphocyte activation**	0.015447404881034767	HLA-G,RASAL3,CLCF1,CDKN1A,TFRC,HLA-A,SHB,,ZBTB1,ABL1,PCK1,NFKBIZ,HLX,HLA-F,HLA-E,RUNX1,SLC7A1,IL12A,TNFSF13B,CAV1,IRS2,HLA-DPB1,FOXO3,TNFSF9,ARID2,NFKBID,PHF10,EFNB2,ZMIZ1,HLA-DPA1,TLR4,CD274,JAK2,ABL2,TNFRSF13C,SOCS1,BCL10,AXL,DPP4,CD320,EPHB2,IL4R,IL15RA,INPP5D,SOX13,KITLG,CARD11,RAG1,PDCD1LG2
**GO:0051962**	**positive regulation of**	0.0154644519211255	NEFL,VEGFA,SPINT1,CLCF1,BAIAP2,TPBG,MAPT,VEGFC,FZD3,SLC7A5,TENM4,ETV5,MAP2K1,,EGR2,MAP6,LIF,EPHB1,SEMA7A,PLXNB3,AGRN,PLXNA3,NGF,,CLSTN1,CRABP

	**nervous system development**		2,WNT2,STK25,,SYNDIG1,ARHGAP32,SOX8,SKIL,TIAM1,NTN1,EPHB2,IL1RAP,PLAG1,SS18L1,NEURL1,AMIGO3,CLSTN2,PLXNA4,LRRC4B,TP73
**GO:0046649**	**lymphocyte activation**	0.01933513301361352	RORA,HLA-G,PRR7,RASAL3,CLCF1,LOXL3,CDKN1A,TFRC,PAG1,HLA-A,FZD7,JAG2,RBPJ,SHB,,CEBPG,JUNB,ZBTB1,NFIL3,MFNG,JMJD6,ABL1,TPD52,EPHB1,PCK1,INHA,NFKBIZ,HLX,ULBP2,WNT4,HLA-F,HLA-E,RUNX1,SLC7A1,F2RL1,IL12A,TNFSF13B,CAV1,DUSP3,IRS2,EGR1,HLA-DPB1,FOXO3,TNFSF9,SH3RF1,ARID2,MAFB,CEACAM1,NFKBID,PHF10,EFNB2,ZMIZ1,HLA-DPA1,CASP3,TLR4,CD274,CDK6,TNFAIP3,JAK2,,ABL2,TNFRSF13C,ULBP1,ARMC5,SOCS1,BCL10,NEDD9,,AXL,DPP4,CAMK4,CD320,EPHB2,IRF4,THEMIS2,IL4R,IL15RA,CBLB,FZD8,EOMES,INPP5D,MICB,SOX13,LFNG,IL20RB,IFNE,FZD5,SLC11A1,KITLG,CARD11,UNC13D,RELB,RAG1,PDCD1LG2,
**GO:0042110**	**T cell activation**	0.02476292912340205	RORA,HLA-G,PRR7,RASAL3,LOXL3,TFRC,PAG1,HLA-A,FZD7,JAG2,SHB,,JUNB,ZBTB1,JMJD6,ABL1,PCK1,NFKBIZ,HLX,WNT4,HLA-E,RUNX1,SLC7A1,F2RL1,IL12A,TNFSF13B,CAV1,DUSP3,EGR1,HLA-DPB1,FOXO3,TNFSF9,SH3RF1,ARID2,MAFB,CEACAM1,NFKBID,PHF10,EFNB2,ZMIZ1,HLA-DPA1,CASP3,CD274,CDK6,JAK2,,ABL2,TNFRSF13C,ARMC5,SOCS1,BCL10,,DPP4,CAMK4,IRF4,IL4R,CBLB,FZD8,EOMES,MICB,SOX13,LFNG,IL20RB,IFNE,FZD5,SLC11A1,KITLG,CARD11,RELB,RAG1,PDCD1LG2,
**GO:0007411**	**axon guidance**	0.024823497179237868	VEGFA,CYFIP2,NOTCH3,EFNA3,FZD3,EGR2,TUBB3,TRIO,ARHGAP35,ECE1,EPHB1,SEMA7A,BOC,PLXNB3,PLXNA3,,EDN1,OBSCN,,NCAM1,SEMA4F,NRXN3,SEMA4B,TUBB2B,EFNB2,NGFR,UNC5B,,PTPRM,FOXD1,SEMA6D,SEMA4G,KLF7,NTN1,EPHB2,NRCAM,SEMA3F,PLXNA4,PTPRO
**GO:0097485**	**neuron projection guidance**	0.024823497179237868	VEGFA,CYFIP2,NOTCH3,EFNA3,FZD3,EGR2,TUBB3,TRIO,ARHGAP35,ECE1,EPHB1,SEMA7A,BOC,PLXNB3,PLXNA3,,EDN1,OBSCN,,NCAM1,SEMA4F,NRXN3,SEMA4B,TUBB2B,EFNB2,NGFR,UNC5B,,PTPRM,FOXD1,SEMA6D,SEMA4G,KLF7,NTN1,EPHB2,NRCAM,SEMA3F,PLXNA4,PTPRO
**GO:1902105**	**regulation of leukocyte differentiation**	0.033988146756928336	KLF10,GPR137B,UBASH3B,HLA-G,FSTL3,LOXL3,SHB,,HLA-B,JUNB,ZBTB1,LIF,ABL1,GPR68,RASSF2,IRF7,PCK1,INHA,NFKBIZ,HLX,RUNX1,LRRC17,FOXO3,TNFSF9,SH3RF1,ARID2,MAFB,CEACAM1,NFKBID,PHF10,ZMIZ1,TLR4,CDK6,,SOCS1,NEDD9,AXL,TRIB1,CAMK4,IRF4,IL4R,IL15RA,INPP5D,SOX13,KITLG,CARD11,RAG1
**GO:0051249**	**regulation of lymphocyte activation**	0.036022063440110955	HLA-G,RASAL3,CLCF1,LOXL3,CDKN1A,TFRC,PAG1,HLA-A,SHB,,JUNB,ZBTB1,ABL1,PCK1,INHA,NFKBIZ,HLX,HLA-F,HLA-E,RUNX1,SLC7A1,IL12A,TNFSF13B,CAV1,DUSP3,IRS2,HLA-DPB1,FOXO3,TNFSF9,SH3RF1,ARID2,CEACAM1,NFKBID,PHF10,EFNB2,ZMIZ1,HLA-DPA1,CASP3,TLR4,CD274,TNFAIP3,JAK2,,ABL2,TNFRSF13C,SOCS1,BCL10,NEDD9,AXL,DPP4,CAMK4,CD320,EPHB2,IRF4,THEMIS2,IL4R,IL15RA,CBLB,INPP5D,SOX13,IL20RB,KITLG,CARD11,RAG1,PDCD1LG2,
**GO:0050870**	**positive regulation of T cell activation**	0.03680480254673512	HLA-G,RASAL3,TFRC,HLA-A,SHB,,ZBTB1,ABL1,PCK1,NFKBIZ,HLX,HLA-E,RUNX1,SLC7A1,IL12A,TNFSF13B,CAV1,HLA-DPB1,FOXO3,TNFSF9,ARID2,NFKBID,PHF10,EFNB2,ZMIZ1,HLA-DPA1,CD274,JAK2,ABL2,TNFRSF13C,SOCS1,BCL10,DPP4,IL4R,SOX13,KITLG,CARD11,RAG1,PDCD1LG2

Supp. Table S6. GO down-regulated genes COMB. vs CTR.

**GO.ID**	**Description**	**FDR**	**Genes**
**GO:0140053**	**mitochondrial gene expression**	1,46E-31	MRPL15,MRPL32,HSD17B10,MRPL24,MRPL52,MRPL3,MRPS27,MRPL20,C1QBP,ELAC2,MRPL1,MRPL16,WARS2,PNPT1,MRPL34,MRPS9,MRPL11,MRPS18C,CHCHD10,MRPL46,MRPS12,CHCHD1,MRPS28,MRPS23,LARS2,GFM1,SARS2,MRPL27,TFB2M,MRPL41,MRPS18B,QRSL1,TRNT1,TRMT5,UQCC2,MRPS14,MRPL47,MRPS30,YARS2,NDUFA7,TRUB2,MRPL4,MRPS15,MRPS17,MRPS35,GFM2,TSFM,MRPL9,MRPL18,MRPS21,MRPL33,MRPL51,MRPL58,MPV17L2,GATB,MRPL30,DARS2,DROSHA,MRPL23,FASTKD2,MRPL54,TRMT61B,MRPS31
**GO:0032543**	**mitochondrial translation**	1,69E-29	MRPL15,MRPL32,MRPL24,MRPL52,MRPL3,MRPS27,MRPL20,C1QBP,MRPL1,MRPL16,WARS2,MRPL34,MRPS9,MRPL11,MRPS18C,MRPL46,MRPS12,CHCHD1,MRPS28,MRPS23,LARS2,GFM1,SARS2,MRPL27,MRPL41,MRPS18B,QRSL1,UQCC2,MRPS14,MRPL47,MRPS30,YARS2,NDUFA7,TRUB2,MRPL4,MRPS15,MRPS17,MRPS35,GFM2,TSFM,MRPL9,MRPL18,MRPS21,MRPL33,MRPL51,MRPL58,MPV17L2,GATB,MRPL30,DARS2,DROSHA,MRPL23,FASTKD2,MRPL54,MRPS31
**GO:0045333**	**cellular respiration**	6,60E-12	SLC25A12,DNAJC15,SLC25A13,CCNB1,ATP5PF,PPARGC1A,DLAT,FH,NDUFS6,NDUFB6,NDUFB1,NDUFA12,CISD1,MYBBP1A,NDUFS8,PNPT1,SUCLG1,SUCLG2,STOML2,COA6,CHCHD10,NDUFB5,OXA1L,NDUFS3,UQCR10,UQCC2,MTLN,NDUFS2,CAT,MRPS36,NDUFB3,DHTKD1,NDUFA7,ATP5F1A,SDHAF4,ETFA,ETFB,ATP5ME,COX7A1,NDUFB10,BLOC1S1,NDUFA9,NDUFS5,ATP5F1E,ATP5PD,SDHD,MSH2,ETFRF1,ME3,MDH1B,OGDHL
**GO:0015980**	**energy derivation by oxidation of organic compounds**	4,58E-11	SLC25A12,DNAJC15,SLC25A13,GYG1,CCNB1,ATP5PF,PPARGC1A,DLAT,FH,NDUFS6,NDUFB6,NDUFB1,NDUFA12,CISD1,MYBBP1A,NDUFS8,POMC,PNPT1,GYG2,SUCLG1,SUCLG2,STOML2,COA6,CHCHD10,NDUFB5,OXA1L,NDUFS3,UQCR10,UQCC2,MTLN,NDUFS2,CAT,MRPS36,NDUFB3,DHTKD1,NDUFA7,ATP5F1A,SDHAF4,ETFA,ETFB,NHLRC1,ATP5ME,COX7A1,NDUFB10,BLOC1S1,NDUFA9,NDUFS5,ATP5F1E,ATP5PD,SDHD,IGF1,MSH2,ETFRF1,,ACADM,ME3,GCK,EPM2A,MDH1B,OGDHL

**GO:0007005**	**mitochondrion organization**	2,04E-10	NDUFAF4,HSD17B10,TMEM126B,GGCT,STAT2,PHB2,NDUFAF2,DNAJC15,TMEM126A,CAMKMT,TOMM6,SLIRP,PPARGC1A,TIMM8A,NDUFB6,MSTO1,NDUFB1,NDUFA12,TOMM40L,PTCD2,BCS1L,TNFSF10,NDUFS8,CIBAR1,AIFM1,RAB29,PNPT1,PARP1,MIPEP,STOML2,HSPD1,TOMM22,TIMM9,CHCHD10,NDUFB5,OXA1L,NDUFS3,TIMM50,SAMM50,DNAJC19,UQCC2,NDUFS2,COX16,NDUFB3,ADCK1,TIMM17A,SDHAF4,CHCHD4,COX7A1,NDUFB10,CHCHD3,SCO1,ACAD9,NDUFA9,NDUFS5,TMEM223,PAM16,CDKN2A,SSBP1,OMA1,ATAD3A,TIMM10,IGF1,FUNDC1,PRIMPOL,MAIP1,TOMM70,NDUFAF5,HGF,,MAP1LC3C,TIMM23B,PDCD5,FMC1,EPM2A,IMMP1L,AURKA,ATP23,PLD6,TYMP
**GO:0009117**	**nucleotide metabolic process**	9,48E-09	NNMT,MTHFD1,DCTPP1,ATP5MC1,ATIC,MCCC2,NAPRT,ACSF2,NQO1,PDE1A,ITPA,SLC25A13,ADCY5,NME1-NME2,ATP5PF,PPARGC1A,DLAT,NDUFS6,NDUFB6,NDUFB1,NDUFA12,NDUFS8,ACAT1,LDHB,PARP9,QPRT,ACSL5,PARP1,SUCLG1,SUCLG2,NUDT16,STOML2,NTHL1,PFAS,NME1,GART,NUDT12,NDUFB5,REXO2,TALDO1,NDUFS3,ACSS1,PMVK,PDE4B,CROT,NDUFS2,NDUFB3,NDUFA7,ATP5F1A,SULT1A3,ATP5ME,AK5,NDUFB10,NDUFA9,DNPH1,NDUFS5,ATP5F1E,PRPS1,ATP5PD,SDHD,ADCY4,CAD,RNASEH2B,,NUDT7,SAMHD1,DLG2,GCK,NUDT17,MCEE,PDK4,MDH1B,ABCC6,TYMP
**GO:0009060**	**aerobic respiration**	1,52E-08	DNAJC15,CCNB1,ATP5PF,DLAT,FH,NDUFS6,NDUFB6,NDUFB1,NDUFA12,NDUFS8,SUCLG1,SUCLG2,STOML2,COA6,CHCHD10,NDUFB5,OXA1L,NDUFS3,UQCR10,UQCC2,NDUFS2,CAT,MRPS36,NDUFB3,DHTKD1,NDUFA7,ATP5F1A,ATP5ME,COX7A1,NDUFB10,BLOC1S1,NDUFA9,NDUFS5,ATP5F1E,ATP5PD,SDHD,MSH2,ME3,MDH1B,OGDHL
**GO:0033108**	**mitochondrial respiratory chain complex assembly**	2,33E-08	NDUFAF4,TMEM126B,NDUFAF2,TMEM126A,NDUFB6,NDUFB1,NDUFA12,BCS1L,NDUFS8,AIFM1,NDUFB5,OXA1L,NDUFS3,SAMM50,UQCC2,NDUFS2,COX16,NDUFB3,SDHAF4,CHCHD4,NDUFB10,SCO1,ACAD9,NDUFA9,NDUFS5,TMEM223,OMA1,NDUFAF5
**GO:0010257**	**NADH dehydrogenase complex assembly**	8,77E-07	NDUFAF4,TMEM126B,NDUFAF2,TMEM126A,NDUFB6,NDUFB1,NDUFA12,BCS1L,NDUFS8,AIFM1,NDUFB5,OXA1L,NDUFS3,NDUFS2,NDUFB3,NDUFB10,ACAD9,NDUFA9,NDUFS5,NDUFAF5
**GO:0032981**	**mitochondrial respiratory chain complex I assembly**	8,77E-07	NDUFAF4,TMEM126B,NDUFAF2,TMEM126A,NDUFB6,NDUFB1,NDUFA12,BCS1L,NDUFS8,AIFM1,NDUFB5,OXA1L,NDUFS3,NDUFS2,NDUFB3,NDUFB10,ACAD9,NDUFA9,NDUFS5,NDUFAF5
**GO:0042776**	**proton motive force-driven mitochondrial ATP synthesis**	0.000002226481379093972	ATP5PF,NDUFS6,NDUFB6,NDUFB1,NDUFA12,NDUFS8,STOML2,NDUFB5,NDUFS3,NDUFS2,NDUFB3,NDUFA7,ATP5F1A,ATP5ME,NDUFB10,NDUFA9,NDUFS5,ATP5F1E,ATP5PD,SDHD
**GO:0015986**	**proton motive force-driven ATP synthesis**	0.0000072049097299461285	ATP5MC1,ATP5PF,NDUFS6,NDUFB6,NDUFB1,NDUFA12,NDUFS8,STOML2,NDUFB5,NDUFS3,NDUFS2,NDUFB3,NDUFA7,ATP5F1A,ATP5ME,NDUFB10,NDUFA9,NDUFS5,ATP5F1E,ATP5PD,SDHD
**GO:0006754**	**ATP biosynthetic process**	0.000013800423022600828	ATP5MC1,SLC25A13,ATP5PF,PPARGC1A,NDUFS6,NDUFB6,NDUFB1,NDUFA12,NDUFS8,PARP1,STOML2,NDUFB5,NDUFS3,NDUFS2,NDUFB3,NDUFA7,ATP5F1A,ATP5ME,NDUFB10,NDUFA9,NDUFS5,ATP5F1E,ATP5PD,SDHD
**GO:0022904**	**respiratory electron transport chain**	0.00002989227895066849	SLC25A12,DNAJC15,SLC25A13,CCNB1,PPARGC1A,NDUFS6,NDUFB6,NDUFB1,NDUFA12,MYBBP1A,NDUFS8,COA6,NDUFB5,NDUFS3,UQCR10,NDUFS2,NDUFB3,NDUFA7,ETFA,ETFB,COX7A1,NDUFB10,NDUFA9,NDUFS5,SDHD,ETFRF1
**GO:0022900**	**electron transport chain**	0.00003919739346895304	SLC25A12,DNAJC15,SLC25A13,CCNB1,PPARGC1A,NDUFS6,NDUFB6,NDUFB1,NDUFA12,MYBBP1A,NQO2,NDUFS8,GFUS,COA6,NDUFB5,NDUFS3,UQCR10,CYB561,NDUFS2,NDUFB3,NDUFA7,ETFA,ETFB,COX7A1,FDX2,NDUFB10,NDUFA9,NDUFS5,SDHD,CYB5A,ETFRF1,FDX1
**GO:0006119**	**oxidative phosphorylation**	0.00009451641813016724	DNAJC15,CCNB1,ATP5PF,NDUFS6,NDUFB6,NDUFB1,NDUFA12,NDUFS8,STOML2,COA6,CHCHD10,NDUFB5,NDUFS3,UQCR10,UQCC2,NDUFS2,NDUFB3,NDUFA7,ATP5F1A,ATP5ME,COX7A1,NDUFB10,NDUFA9,NDUFS5,ATP5F1E,ATP5PD,SDHD,MSH2
**GO:0046034**	**ATP metabolic process**	0.0004187902157895431	ATP5MC1,SLC25A13,ATP5PF,PPARGC1A,NDUFS6,NDUFB6,NDUFB1,NDUFA12,NDUFS8,PARP1,STOML2,NDUFB5,NDUFS3,NDUFS2,NDUFB3,NDUFA7,ATP5F1A,ATP5ME,AK5,NDUFB10,NDUFA9,NDUFS5,ATP5F1E,ATP5PD,SDHD,ABCC6
**GO:0000959**	**mitochondrial RNA metabolic process**	0.002966922666309638	HSD17B10,SLIRP,ELAC2,WARS2,PNPT1,CHCHD10,SARS2,TFB2M,TRNT1,TRMT5,YARS2,DARS2,FASTKD2,TRMT61B
**GO:0070585**	**protein localization to mitochondrion**	0.007686376568128779	DNAJC15,TOMM6,TIMM8A,TOMM40L,BCS1L,AIFM1,MIPEP,HSPD1,TOMM22,TIMM9,OXA1L,TIMM50,SAMM50,DNAJC19,TIMM17A,CHCHD4,PAM16,TIMM10,MAIP1,TOMM70,TIMM23B,PDCD5,IMMP1L
**GO:0006626**	**protein targeting to mitochondrion**	0.009327303317721268	DNAJC15,TOMM6,TIMM8A,TOMM40L,AIFM1,MIPEP,HSPD1,TOMM22,TIMM9,TIMM50,SAMM50,DNAJC19,TIMM17A,CHCHD4,PAM16,TIMM10,TOMM70,TIMM23B,PDCD5,IMMP1L
**GO:0007007**	**inner mitochondrial membrane organization**	0.041595731878398295	TIMM8A,CIBAR1,TIMM9,CHCHD10,SAMM50,ADCK1,CHCHD3,OMA1,TIMM10,MAIP1,TOMM70
